# Genetic variation at the MHC *DRB1* locus is similar across Gunnison's prairie dog (*Cynomys gunnisoni*) colonies regardless of plague history

**DOI:** 10.1002/ece3.2077

**Published:** 2016-03-16

**Authors:** Kacy R. Cobble, Katy J. Califf, Nathan E. Stone, Megan M. Shuey, Dawn N. Birdsell, Rebecca E. Colman, James M. Schupp, Maliha Aziz, Roger Van Andel, Tonie E. Rocke, David M. Wagner, Joseph D. Busch

**Affiliations:** ^1^ Center for Microbial Genetics and GenomicsNorthern Arizona UniversityPO Box 4073FlagstaffArizona86011USA; ^2^ Translational Genomics Research Institute North3051 W. Shamrell Blvd #106FlagstaffArizona86001USA; ^3^ University of California BerkeleyMC 7150BerkeleyCalifornia94720USA; ^4^ United States Geological SurveyNational Wildlife Health Center6006 Schroeder RoadMadisonWisconsin53711USA

**Keywords:** *Cynomys gunnisoni*, MHC‐*DRB1*, Plague, Prairie dog, *Yersinia pestis*

## Abstract

*Yersinia pestis* was introduced to North America around 1900 and leads to nearly 100% mortality in prairie dog (*Cynomys* spp.) colonies during epizootic events, which suggests this pathogen may exert a strong selective force. We characterized genetic diversity at an MHC class II locus (*DRB1*) in Gunnison's prairie dog (*C. gunnisoni*) and quantified population genetic structure at the *DRB1* versus 12 microsatellite loci in three large Arizona colonies. Two colonies, Seligman (SE) and Espee Ranch (ES), have experienced multiple plague‐related die‐offs in recent years, whereas plague has never been documented at Aubrey Valley (AV). We found fairly low allelic diversity at the *DRB1* locus, with one allele (DRB1*01) at high frequency (0.67–0.87) in all colonies. Two other *DRB1* alleles appear to be trans‐species polymorphisms shared with the black‐tailed prairie dog (*C. ludovicianus*), indicating that these alleles have been maintained across evolutionary time frames. Estimates of genetic differentiation were generally lower at the MHC locus (*F*_ST_ = 0.033) than at microsatellite markers (*F*_ST_ = 0.098). The reduced differentiation at *DRB1* may indicate that selection has been important for shaping variation at MHC loci, regardless of the presence or absence of plague in recent decades. However, genetic drift has probably also influenced the *DRB1* locus because its level of differentiation was not different from that of microsatellites in an *F*_ST_ outlier analysis. We then compared specific MHC alleles to plague survivorship in 60 *C. gunnisoni* that had been experimentally infected with *Y. pestis*. We found that survival was greater in individuals that carried at least one copy of the most common allele (DRB1*01) compared to those that did not (60% vs. 20%). Although the sample sizes of these two groups were unbalanced, this result suggests the possibility that this MHC class II locus, or a nearby linked gene, could play a role in plague survival.

## Introduction

Infectious diseases can play an important role in shaping genetic variation and fitness in free‐living populations (Anderson and May 1979; Keller and Waller 2002; Keesing et al. 2010). For example, diseases may decrease allelic variation at immune system loci via selective sweeps (de Groot et al. 2002; Carnero‐Montoro et al. 2012) and reduce variation at neutral loci as a result of genetic bottlenecks (Maruyama and Fuerst 1985; Luikart and Cornuet 1998). Conversely, a cyclic pattern of rare allele advantage under pressure by coevolving pathogens (an evolutionary arms race) should result in the maintenance of a greater number of alleles over time due to balancing selection (Bodmer 1972; Potts and Wakeland 1990; Slade and McCallum 1992). Empirical evidence from a growing number of species suggests that increased genetic diversity at neutral and adaptive loci is associated with reduced susceptibility of individuals to infectious diseases and parasites (O'Brien and Evermann 1988; Siddle et al. 2007; Altermatt and Ebert 2008). Therefore, characterizing extant genetic diversity, particularly at functionally relevant immune system genes, can contribute to a more comprehensive understanding of the complex dynamics between genetic diversity and disease resistance in free‐living populations. Among populations of conservation concern, introduced diseases are considered one of the top extinction dangers (Altizer et al. 2003). Thus, understanding the impact of infectious diseases in wild systems is critical for species management and protecting human public health (Daszak 2000).

An introduced disease organism with significance for many rodents in western North America is *Yersinia pestis*, the causative agent of plague. This bacterial pathogen originated in Asia and was introduced to several U.S. ports from China around 1900, at which time it became established in commensal rodents and fleas in San Francisco (Link 1955; Gage and Kosoy 2005). The pathogen soon jumped to native rodents, especially colonial ground squirrels (Eskey and Haas 1940), and spread eastward from California. Plague began affecting prairie dogs (*Cynomys* spp.) in the 1930s and led to major population reductions in the five species found in western North America (Hoogland et al. 2004). Prairie dog decline has become a critical conservation concern for black‐footed ferrets (*Mustela nigripes*) because they are highly specialized predators of prairie dogs that are completely dependent on large persistent colonies for survival (Biggins et al. 2006).

Given the repeated occurrence of plague epizootics in the western United States and their high mortality in prairie dog populations, it is reasonable to infer that plague has been a strong selective force on prairie dogs and other rodents over the past 80 years (Thomas et al. 1988). All prairie dog species appear to be highly susceptible to *Y. pestis*, and mortality levels reach nearly 100% in colonies of Gunnison's prairie dog (*C. gunnisoni*) (Cully 1997; Cully et al. 1997), Utah prairie dog (*C. parvidens*) (Cully and Williams 2001), and black‐tailed prairie dog (*C. ludovicianus*) (Pauli et al. 2006; Cully et al. 2010). White‐tailed prairie dog (*C. leucurus*) colonies seem to experience a lower mortality rate (Anderson and Williams 1997; Griffin et al. 2010), possibly because they are less social than other prairie dogs and do not spread the pathogen as efficiently (Gasper and Watson 2001). Gunnison's prairie dog appears to be particularly susceptible to local colony extirpations resulting from plague (Eskey and Haas 1940; Girard et al. 2004), and up to 70% of monitored *C. gunnisoni* colonies in the state of Arizona were extirpated between the early 1980s and 2001 (Wagner et al. 2006). The range of *C. gunnisoni* lies in the Four Corners area, a region of the southwestern United States that has been a major focus of plague outbreaks in the past century (Ari et al. 2010).

Two recent experimental challenge studies in *C. gunnisoni* and *C. ludovicianus* suggest that some resistance to *Y. pestis* may exist (Rocke et al. 2012; Busch et al. 2013). Survival of wild‐caught *C. ludovicianus* collected in plague‐endemic areas of Colorado and Texas (50% and 60% survival, respectively) was much higher during exposure to the fully virulent *Y. pestis* strain CO92 than individuals originating from a colony in western South Dakota (5% survival) that had not previously experienced plague (Rocke et al. 2012). A similarly high survival rate (50–70%) was found during an experimental challenge with *Y. pestis* CO92 in *C. gunnisoni* from two populations in Arizona (Espee Ranch and Aubrey Valley) (Busch et al. 2013). A strong antibody response does not appear to be the primary mechanism of survival in either species and suggests the possibility of a genetic component for plague resistance.

**Figure 1:**
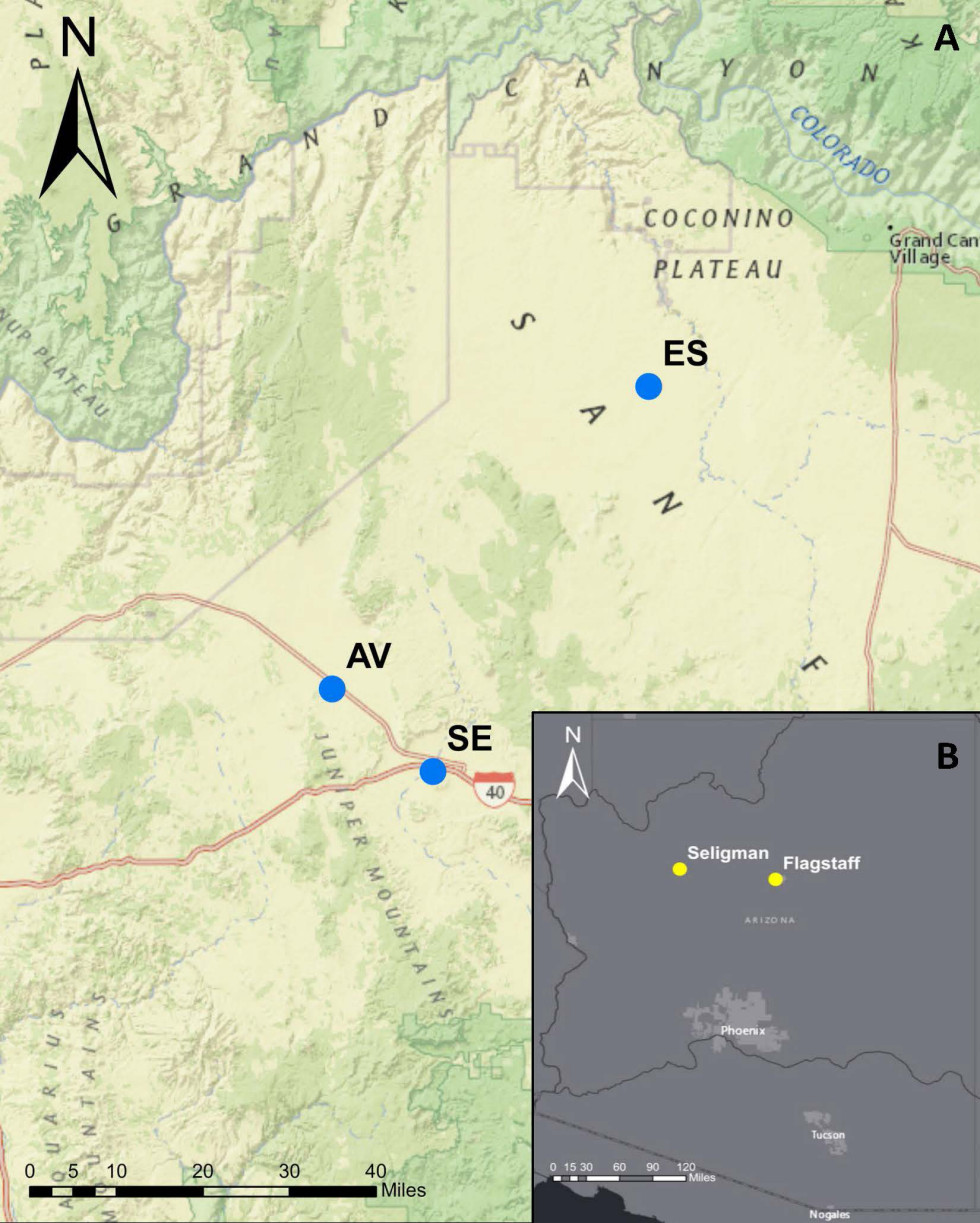
Map of the three *Cynomys gunnisoni* colonies from this study. (A) Location of the three study colonies; AV = Aubrey Valley; SE = Seligman; ES = Espee Ranch. (B) Inset map showing the study area in Arizona. The AV and ES colonies (66 km apart) are reintroduction sites for black‐footed ferrets (*Mustela nigripes*). Additional details of collection locations are available in a previous publication (Busch et al. 2011).

Both the Aubrey Valley (AV) and Espee Ranch (ES) populations are closely monitored because they are release sites for black‐footed ferrets (Fig. 1). Plague is known to occur at ES, and the most recent outbreaks occurred in 2009 (Busch et al. 2013) and 2014 (T. Rocke, *unpublished data*). In contrast, the AV population has not experienced any observable mortality from plague, even during years when plague epizootics affected nearby colonies (Van Pelt 1995). In 1996, plague occurred within just 6–10 km of AV at an adjacent colony complex near Seligman, AZ (the SE population). Despite apparent differences in plague occurrence, both AV and ES prairie dogs showed higher survivorship than expected during experimental challenge (Busch et al. 2013), which suggests that many individuals in both colonies may have some level of resistance to the disease.

A recent study of plague in Madagascar has suggested that multiple MHC regions may be under selection in black rat (*Rattus rattus*) populations that are regularly exposed to endemic *Y. pestis* (Tollenaere et al. 2012). Class I and II MHC genes are among the most polymorphic genes in the vertebrate genome (Klein and Figueroa 1986; Edwards and Hedrick 1998), and variability in this region often reflects evolutionarily relevant processes (Sommer 2005). The second exon of the class II *DRB* gene often bears a signature of selection because it comprises a substantial portion of the antigen‐binding region of this molecule (Klein et al. 1993). Variation in MHC genes has been linked to individual fitness in the presence of parasites (Paterson et al. 1998; Thoss et al. 2011; Kloch et al. 2013), but little is known about the specific contribution made by the class II *DRB* locus during *Y. pestis* infection.

In this study, we investigated patterns of genetic variation in *C. gunnisoni* colonies where plague is known to occur repeatedly, ES and SE, and the AV colony, where plague appears to be absent. We compared genetic structure at microsatellite markers with that of the MHC class II *DRB*1 locus. Other studies have used comparisons of neutral markers versus MHC genes to detect recent selection acting on the MHC gene region (Boyce et al. 1997; Landry and Bernatchez 2001; Worley et al. 2006). Assuming that plague is a strong selective pressure on *C. gunnisoni* populations and that this disease has a similar effect on all colonies (e.g., nearly 100% mortality) when it is present, we hypothesized that this MHC locus would exhibit appreciably lower levels of genetic differentiation (*F*_ST_) than observed at microsatellite markers. Neutral genetic markers should show greater levels of genetic structure due to population fluctuations and the increased strength of genetic drift on small populations. Likewise, we predicted that the AV population will have greater variation at the MHC locus, because this population has not been subjected to the multiple plague events that could result in reduced diversity at other colonies (ES and SE). We also generated MHC genotypes of all *C. gunnisoni* individuals from the previous experimental plague challenge study (Busch et al. 2013) to explore the association of specific *DRB1* alleles with individual survivorship.

## Material and Methods

### Sample collection and DNA extraction

We sampled three wild populations of *C. gunnisoni* in northern Arizona to evaluate genetic diversity at the MHC class II *DRB1* locus and 12 microsatellite repeat markers. Multiple tissues such as spleen, liver, lung, muscle, skin, and blood were collected from 94 prairie dogs. Animals were collected during two separate sampling sessions (Appendix 1). In the first sampling session (2005–2006), 34 prairie dogs were sampled for a previous study (Busch et al. 2011) using animals shot by local hunters at Aubrey Valley (AV2006, *n *=* *16) and a neighboring colony at Seligman, Arizona (SE, *n *=* *18). In the second session (2009), prairie dogs were live‐trapped from two colonies where black‐footed ferrets have been released in Arizona: AV (*n *=* *30) and Espee Ranch (ES) (*n *=* *30). The 60 animals from 2009 are designated as AV_C_ or ES_C_ in this study because they were taken into captivity and used in a plague challenge experiment for *C. gunnisoni* (Busch et al. 2013). Important details of survivorship (*Y. pestis* dose, fate, sex, age, etc.) for these 60 individuals are provided in Appendix 1. All tissue samples, including those collected in the field, were quickly frozen in liquid nitrogen and stored at −80°C prior to DNA or RNA extraction. We also made use of skin samples from black‐tailed prairie dogs (*C. ludovicianus*) live‐trapped in Phillips County, Montana after a plague epizootic in 2008 (generously donated by Randy Matchett from the Charles M. Russell National Wildlife Refuge). The *C. ludovicianus* samples were used as a species comparison for our initial description of MHC *DRB1* alleles from *C. gunnisoni*. All work was approved by the Northern Arizona University Institutional Animal Care and Use Committee under protocol 05‐005.

Spleen or muscle samples were used to extract genomic DNA (gDNA) from *C. gunnisoni*. Tissues were disrupted with bead milling for 20 sec following a previous protocol (Allender et al. 2004), and gDNA was extracted using the Qiagen DNeasy Blood and Tissue Kit (Qiagen, Valencia, CA) according to the manufacturer's instructions. DNA samples were diluted to 20 ng/*μ*L working stocks for downstream PCR methods.

### MHC sequencing

We used two sequencing‐based approaches to initially isolate and characterize the MHC class II *DRB1* gene, which has not yet been described for any prairie dog species. First, we used a traditional approach to amplify, clone, and sequence the exon 2 fragment of the *DRB1* locus using *HLA* primers GH46F/GH50R (Erlich and Bugawan 1990) (Appendix 2). PCRs were carried out in 10 *μ*L volumes containing the following reagents (given in final concentrations): 20–40 ng of gDNA template, 1X PCR buffer, 2 mM MgCl_2_, 0.2 mM dNTPs, 2 U Platinum^®^ *Taq* DNA polymerase (Invitrogen, Carlsbad, CA), and 0.2 *μ*M of each primer. PCRs were thermocycled according to the following conditions: 95°C for 10 min to release the Plat *Taq* ^®^ antibody, followed by 35 cycles of 94°C for 15 sec, 65°C for 20 sec, 72°C for 30 sec, and a final extension step of 72°C for 5 min. PCR products were electrophoresed on a 2% agarose gel for 1 h at 100V. If amplification was faint for an individual, additional PCRs were obtained before cleaning with a QiaQuick PCR Purification Kit (Qiagen, Valencia, CA).

To determine the specific *DRB1* alleles present in heterozygous individuals, purified PCR product was ligated into the pGEM‐T vector (Promega, Madison, WI) and transformed into *Escherichia coli* JM109 cells. Cells were plated onto ampicillin/IPTG/X‐Gal agar and cultured at 37°C for 18 h before picking positive clones (white or light blue in color). PCR screens were performed on 24 colonies using the same conditions as for the initial PCR, except that primers M13F and R were used at an annealing temperature of 55°C. Eighteen positive clones from each sample were sequenced to ensure detection of each individual allele (Babik 2010). Sequencing reactions were performed with M13 primers using a BigDye^®^ v3.1 Cycle Sequencing Kit (Applied Biosystems, Foster City, CA), and the products were separated on an ABI3130 automated sequencer. Sequences were aligned using the Lasergene software package (DNASTAR, Madison, WI).

Our second approach to MHC class II gene discovery in *C. gunnisoni* used a Roche 454 platform to pyrosequence PCR amplicons using GH46F/GH50R primers modified with sequencing adaptors and bar codes (Appendix 2). These tailed primers were used in a 10‐*μ*L PCR with the following conditions: 40 ng gDNA, 1 U Platinum^®^ *Taq* DNA polymerase, 1X PCR buffer, 2 mM MgCl_2_, 0.2 mM dNTPs, and 0.4 *μ*M each primer. A stringent touchdown procedure was required to obtain clean amplification of the *DRB1* gene. Thermocycler conditions started at 95°C for 10 min, followed by eight cycles of 94°C for 45 sec, 66°C for 30 sec, and 72°C for 30 sec. In the next ten cycles, we decreased the annealing temperature (*T*_a_) by 0.4°C per cycle (decrement from 66°C to 62.4°C). The *T*_a_ was then kept at 62°C for the remaining 17 cycles (35 cycles total), with a final extension of 72°C for 10 min. We pooled equimolar amounts of these PCR products and shipped them to the Genome Resource Center at the University of Maryland for 454 sequencing. Raw 454 sequence reads for each prairie dog were sorted by bar code and clustered into unique alleles. These allele clusters were aligned in SeqMan Pro (DNASTAR, Madison, WI) to generate consensus sequences for each prairie dog.

### Validation of *DRB* expression

It is important to validate the expression of newly described *DRB1* alleles, especially in nonmodel organisms. Therefore, we used a Total RNA Purification Kit (Norgen Biotek, Thorold, ON) to extract RNA from approximately 40 mg of spleen tissue from a subset of *C. gunnisoni* (*n *=* *10); these ten validation individuals are highlighted in the first column of Appendix 1 with a “^V^” notation. We treated the total RNA with amplification grade DNase I (Invitrogen, Grand Island, NY) to degrade possible residual gDNA that may have been present in the RNA extractions. To confirm the absence of gDNA, we diluted the RNA 1/10 with molecular grade H_2_O for PCR and used the exon 2 primers GH46F/GH50R (Appendix 2). *Taq* polymerase is incompatible with RNA templates, and therefore, PCR failure indicated the absence of gDNA. Positive control reactions with *C. gunnisoni* gDNA were included, and all reactions were electrophoresed on 2% agarose as described above to validate the degradation of DNA.

Undiluted total RNA samples were then used as templates to synthesize cDNA in 10 *μ*L reactions using Smartscribe^™^ reverse transcriptase (Clonetech, Mountain View, CA) and two oligos: 3′ SMART CDS Primer II (5′‐AAGCAGTGGTATCAACGCAgtacTTTTTTTTTTTT TTTTTTTTTTTTTTTTTTVN‐3′) and SMART II G‐linker (5′‐AAGCAGTGGTATCAAC GCAgtacGCGGG‐3′). The incubation time for first strand synthesis was extended to 90 min, and the reaction product was diluted with 40‐*μ*L molecular grade H_2_O. Resulting cDNA was quantified on a NanoDrop 8000 spectrophotometer (Thermo Scientific, Waltham, MA) and diluted to 20–40 ng/*μ*L for PCR. Successful cDNA synthesis was confirmed using the exon 2 primer PD1*DRB*_F (5′‐CGTTTCCTGGAGCAAGTTTCACATG‐3′) and a reverse primer anchored in exon 3, *DRB*exon3‐R2 (5′‐AGACCAGGAGGTTGTGGTGCTG‐3′) (Appendix 2). PCRs were carried out in 10 *μ*L volumes containing the following reagents (given in final concentrations): 20–40 ng of cDNA template, 1X PCR buffer, 2 mM MgCl_2_, 0.2 mM dNTPs, 0.2 *μ*M of each primer, 1 U Platinum^®^ *Taq* DNA polymerase, and 0.2 *μ*M of each primer. PCRs were thermocycled according to the following conditions: 95°C for 10 min to release the Plat *Taq* ^®^ antibody, followed by 35 cycles of 94°C for 15 sec, 65°C for 20 sec, and 72°C for 30 sec, with a final extension step of 72°C for 10 min. PCR products were electrophoresed on a 2% agarose gel for 1 h at 100V. Amplicons were cloned into the pGEM‐T vector (Promega) and sequenced as described above. Each unique allele that was validated at this stage was named according to conventions for nonmodel species (e.g., *MhcCygu*‐DRB1*01 and *MhcCygu*‐DRB1*02) (Klein et al. 1990).

In the process of sequencing exons 2 and 3 from cDNA, we discovered that certain alleles (*Cygu*‐DRB1*02, *Cygu*‐DRB1*05, and *Cygu*‐DRB1*06) had accumulated multiple single nucleotide polymorphisms (SNPs) in the exon 2 priming site for GH50R, which was originally designed for human *DRB* sequences (Appendix 3). The accumulation of up to six SNPs in these alleles created an amplification bias that led to allelic dropout in standard PCR. Therefore, we designed and validated two new reverse primers (*CyguDRB1*_exon2_R1: 5′‐CTCTCCTCTCCACAGTGAAGCTCTCA‐3′ and *CyguDRB1*_exon2_R2: 5′‐CTCTCCGCTCCACAGCGAAGCTCTCA‐3′) that, when combined and used with GH46F, will allow unbiased amplification of all six *MhcCygu*‐*DRB1* alleles (PCR conditions in Appendix 2).

### Quantitative PCR assays for MHC

To provide rapid and accurate identification of all known *DRB1* alleles we discovered in *C. gunnisoni*, we designed six quantitative PCR (qPCR) assays. Briefly, the allele typing process requires three steps (all methods provided in Appendix 2):

1. In the first step, four assays are used to amplify all six known alleles from this study. Alleles *Cygu*‐DRB1*02, *Cygu*‐DRB1*03, and *Cygu*‐DRB1*04 can be unambiguously identified with these four qPCR assays. However, alleles *Cygu*‐DRB1*01, *Cygu*‐DRB1*05, and *Cygu*‐DRB1*06 require 1–2 additional assays to accurately score the allele.
2. Next, the subset of individuals that appear to have at least one *Cygu*‐DRB1*01 allele are tested with an assay to distinguish allele 01 from 05 or 06.
3. If needed, a third PCR with competitive forward primers is used to unambiguously resolve alleles 05 and 06.

The specific PCR outcomes expected for each diploid genotype (21 unique combinations) are described in Appendix 4. These assays were first validated on cDNA, then run against the gDNA of all 94 prairie dogs and compared to the genotypes scored from Sanger and 454 sequences (Appendix 1).

### MHC analyses

To assess genetic diversity at the MHC‐*DRB1* locus within these *C. gunnisoni* populations, we calculated allele frequencies, observed heterozygosity (*H*_O_), expected heterozygosity (*H*_E_), and tested for Hardy–Weinberg equilibrium using a Markov Chain implemented in FSTAT 2.9 (Goudet 1995, 2001). This same program was used to estimate the fixation index, *F*_ST_, for a global mean and all possible pairwise comparisons. Significance testing in FSTAT used 1000 permutations per pairwise comparison. Because we had two population samples from AV separated by three years, the 16 prairie dogs collected in 2006 (AV2006) were excluded in all calculations that produced a mean value (e.g., mean *H*_O_ and global *F*_ST_).

**Figure 2:**
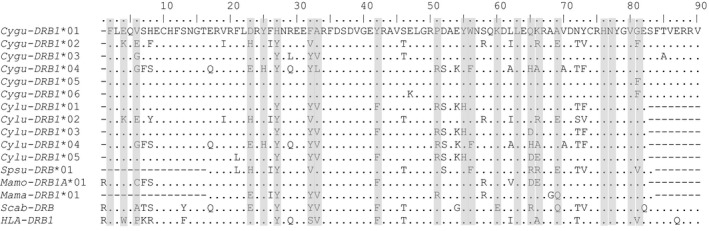
Translated amino acid alignment of MHC‐*DRB1* alleles from Gunnison's prairie dog, *Cynomys gunnisoni* (*MhcCygu*‐*DRB1*, alleles 01–06: GenBank accessions KU695893‐KU695896 and KR338362‐KR338363) and black‐tailed prairie dog, *C. ludovicianus* (*MhcCylu‐DRB1*, alleles 01–05: KR338364‐KR338368). *Dots* represent identity to the *Cygu*‐DRB*01 amino acid sequence at the top. *Dashes* indicate missing data. *Shaded positions* show putative antigen‐binding sites (ABS) after (Yuhki et al. 2008). None of the amino acid sites are predicted to be under positive selection in either prairie dog species. Other ground squirrels are included in the alignment, including spotted suslik (*Spermophilus suslicus*) *Spsu*‐*DRB*: EF569186 (Biedrzycka and Radwan 2008; Biedrzycka et al. 2011); Eastern woodchuck (*Marmota monax*) *Mamo*‐*DRB1*A: KJ675569 (Moreno‐Cugnon et al. 2015); European marmot (*Marmota marmota*) *Mama‐DRB*: JQ837902 (Kuduk et al. 2012), Abert's squirrel (*Sciurus aberti*) *Scab*‐*DRB*: AAA42356; and human (*Homo sapiens*) *HLA*‐*DRB*: NP_002115.

The MHC‐*DRB1* allele sequences found in *C. gunnisoni* were analyzed for evidence of selection acting on the MHC. To do so, our six *MhcCygu*‐*DRB1* allele sequences were aligned with a human sequence, translated to amino acids (Fig. 2), and conserved sites and putative antigen‐binding sites (ABS) were inferred from the human sequence, in accordance with previous studies (Kaufman et al. 1994; Bondinas et al. 2007; Yuhki et al. 2008). We calculated rates of synonymous (*d*_S_) and nonsynonymous (*d*_N_) substitutions per site at 89 amino acid positions and the 21 putative ABS using the Nei‐Gojobori method and applying a Jukes–Cantor correction for multiple substitutions at a site (Nei and Gojobori 1986) in DnaSP version 5 (Librado and Rozas 2009). A *Z*‐test was computed in MEGA v. 6.0.6 to test for positive selection acting on the second exon of this *DRB1* locus (Tamura et al. 2013). We also implemented maximum likelihood and Bayesian methods to test for positive selection using PAML 4.8 (Yang 1997, 2007), as inferring selection from distance‐based measures of *d*_N_/*d*_S_ has been shown to be problematic (Kryazhimskiy and Plotkin 2008; Wolf et al. 2009). A full description of model parameters and tests is provided in Appendix 5.

To further explore the evolutionary history of these alleles, we included five MHC‐*DRB1* alleles isolated from black‐tailed prairie dogs (*C. ludovicianus*). These *MhcCylu‐DRB1* alleles were discovered by PCR amplicon sequencing, but their expression has not been validated at this time. All statistical analyses for the *C. ludovicianus* genetic data were performed as described above for *C. gunnisoni*. A maximum likelihood phylogeny was constructed for the *DRB1* locus of both species of prairie dog using MEGA 6.0.6. The best nucleotide substitution model (AICc = 4081) was found to be Jukes–Cantor with a gamma distribution (JC + G). Confidence estimates for nodes on the tree were generated from 1000 bootstrap replicates.

Because recombination within sequences can lead to the false identification of positive selection (Anisimova et al. 2003), we tested our allele sequences for evidence of recombination in both prairie dog species using three commonly used measures of linkage disequilibrium (*r* ^2^, D', and G4) in the program PERMUTE (Wilson and McVean 2006).

### Microsatellite marker comparisons

To establish a baseline level of genetic variation among the three prairie dog populations, we genotyped all individuals using a set of 12 microsatellite markers (Appendix 6) that were originally developed for black‐tailed prairie dogs (Jones et al. 2005) and ground squirrels (May et al. 1997; Stevens et al. 1997). All details of PCR amplification and multiplexing are provided in Appendix 6. Microsatellite alleles were electrophoresed on an AB3130 sequencer and scored with GENEMAPPER v4.0 software (Applied Biosystems, Foster City, CA). All individuals were run twice at all markers, and no genotyping errors were observed.

To assess neutral genetic structure of the study populations, we calculated *H*_O_ and allele frequencies of our microsatellite data using the program GENALEX 6.4. An exact test for Hardy–Weinberg equilibrium using a Markov Chain was implemented in FSTAT 2.9. Global and pairwise estimates of *F*_ST_ were obtained using 1000 permutations per pairwise comparison in FSTAT. A global estimate of *F*_ST_ from the *DRB1* MHC locus was compared to the global 95% confidence interval (CI) from microsatellite markers.

A second point of comparison was made between microsatellite loci and the *DRB1* locus using the software fdist2 (Beaumont and Nichols 1996). First, a neutral distribution for microsatellite loci was generated, and then outlier loci were removed sequentially from the analysis, starting with those furthest from the 95% CI contour line. fdist2 was rerun iteratively with an adjusted mean *F*_ST_ until all loci followed a neutral distribution. Loci that fell outside of the 95% CI of the neutral distribution were considered to be putatively non‐neutral outliers under positive selection. fdist2 was run with the following parameters: 30 samples per population, four populations, and an expected *F*_ST_ of 0.083 between all populations. The model was run for 50,000 iterations, and the 95% CI of the neutral distribution was calculated. To investigate the relative strength of demographic versus selective effects on MHC allele frequency distributions, we used both neutral microsatellite loci and the adaptive MHC locus to estimate genetic relationships among these populations. fdist2 was rerun without the presumptively non‐neutral microsatellites as described above, and with an adjusted *F*_ST_ of 0.045, as determined from the iterative initial runs of the model. The 95% CI of the neutral distribution was generated and plotted against the pairwise *F*_ST_ values of microsatellites and the MHC *DRB1* locus to determine whether the MHC *DRB1* locus conformed to neutral expectations simulated in fdist2.

Because most *C. gunnisoni* colonies have experienced plague in recent years (including ES and SE), we tested for evidence of genetic bottlenecks in all four population samples. We used the 12 microsatellite loci in the program bottleneck (Piry et al. 1999) to test for heterozygosity excess (Cornuet and Luikart 1996) or a mode shift in the frequency of rare alleles (Luikart et al. 1998). We ran the heterozygosity excess method under two different mutation models, the stepwise mutation model (SMM) and infinite allele model (IAM), and tested significance with the Wilcoxon signed‐rank test to evaluate deviations from a 50:50 ratio of markers with heterozygosity deficiency:excess.

### Survival analyses of *Y. pestis* challenge

We used survival data gathered from 60 *C. gunnisoni* individuals from a previously published *Y. pestis* challenge study (Busch et al. 2013) to determine whether survival in prairie dogs from ES_C_ and AV_C_ was associated with MHC heterozygosity and/or the presence/absence of specific *DRB1* alleles. Distributions of the Kaplan–Meier survival curves for the two populations were compared using the logrank test (Harrington and Fleming 1982) in the “survival” package in R (R Core Team RCT 2013).

## Results

### MHC variation

The *DRB1* exon 2 locus that we characterized in *C. gunnisoni* appears to be a single‐copy, classically expressed MHC gene with the potential to be shaped by natural selection. We detected six unique alleles (MhcCygu‐DRB1*01 to 06: GenBank accessions KU695893–KU695896 and KR338362–KR338363) among the 94 *C. gunnisoni* analyzed in this study (Appendix 1). BLAST comparisons against the GenBank database yielded close similarity to MHC class II *DRB1* sequences from other ground squirrels, especially the spotted suslik, *Spermophilus suslicus* (Biedrzycka and Radwan 2008). At the *C. gunnisoni* exon 2 locus, we recovered a maximum of two alleles per individual and genotype frequencies were in Hardy–Weinberg equilibrium for all four population samples. This result provides compelling evidence that the *C. gunnisoni* exon 2 locus is a single copy. We successfully validated expression of all six alleles using spleen mRNA from a subset of 10 *C. gunnisoni*. Each exon 2 allele is an open‐reading frame (ORF) that encodes a class II beta subunit molecule with no insertions, deletions, or stop codons (Appendix 3). The translated amino acid alignment of this exon 2 locus (Fig. 2) has a combination of variable and conserved sites expected for a classical *DRB1* locus (Kaufman et al. 1994; Bondinas et al. 2007; Yuhki et al. 2008). Together, these lines of evidence provide robust validation that this class II gene is a fully functional MHC gene in *C. gunnisoni*.

Alleles 05 and 06 were rare and occurred in just nine heterozygous individuals (9.6% of the 94), marked with a superscripted “^m^” in Appendix 1. Both the Sanger and 454 sequencing platforms missed every instance of these two alleles, and we did not discover the extent of the allelic dropout problem until all 94 individuals had been screened with our six qPCR assays. The two new reverse primers that we designed (*CyguDRB1*_exon2_R1 and *CyguDRB1*_exon2_R2) at the extreme 3′ end of exon 2 will robustly amplify all six *DRB1* alleles in *C. gunnisoni* (Appendix 2).

Contrary to our prediction, the AV population did not exhibit greater variation at the MHC *DRB1* locus when compared to ES or SE (the two plague sites). In fact, the greatest number of alleles (*n *=* *6) was found at ES. There were only modest differences in allele frequency among these colonies (Appendix 7). Allele *MhcCygu*‐DRB*01 was by far the most common allele, with a frequency of 67–86%. It was also the only allele shared by all three populations. Three of the six alleles (04, 05, and 06) were fairly rare, with frequencies < 0.1. The level of *H*_O_ in this MHC locus ranged from 0.27 to 0.57 (mean *H*_O_ = 0.38 ± 0.1) and was virtually the same as *H*_E_ (0.25–0.52) (Appendix 6). *H*_O_ was not significantly different among the population samples after excluding AV2006 to avoid using redundant samples from AV (Kruskal–Wallace *χ* ^2^ = 3, df = 3, *P *>* *0.1).

**Figure 3:**
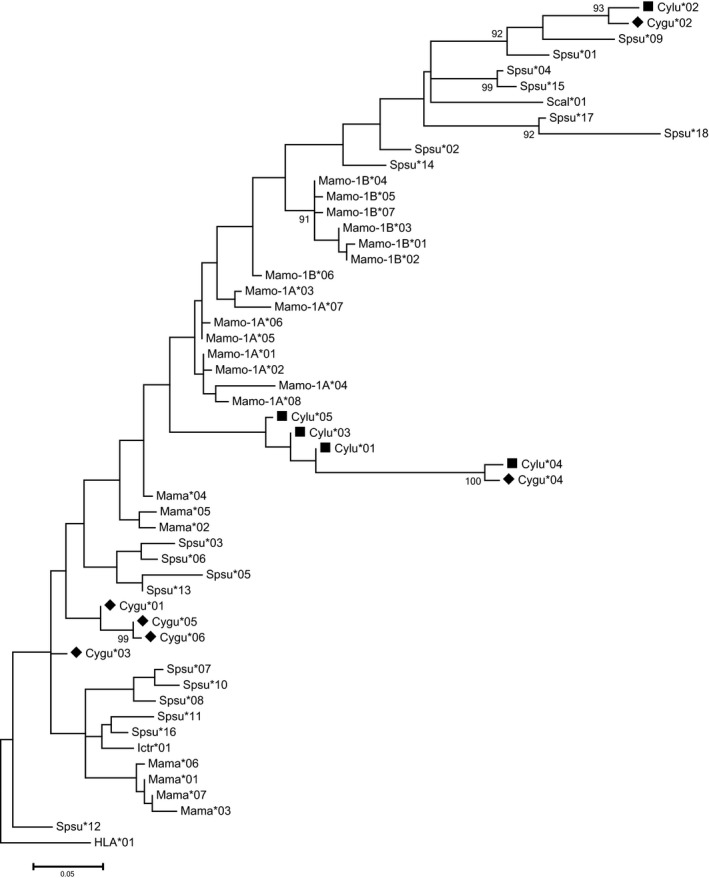
Maximum likelihood tree of MHC‐*DRB1* alleles based on exon 2 nucleotide sequence. Diamonds (♦) denote Gunnison's prairie dog, *Cynomys gunnisoni*, and squares (■) denote black‐tailed prairie dog, *C. ludovicianus*. Nodes with bootstrap confidence values ≥90% are labeled, and the scale bar units are single nucleotide polymorphisms (SNPs). GenBank accession numbers include KU695893‐KU695896 and KR338362‐KR338363 (*MhcCygu*‐*DRB1* alleles 01–06); KR338364‐KR338368 (*MhcCylu‐DRB1* alleles 01–05); JQ837902‐JQ837908 (European marmot (*Marmota marmota*) alleles Mama*01–07); KJ675569‐KJ675576 (Eastern woodchuck (*Marmota monax*) alleles *Mamo*‐1A*01–08) and KJ675577‐KJ575583 (alleles *Mamo*‐1B*01–07); EF569186‐EF569200 and HM461912‐HM461913 (spotted suslik (*Spermophilus suslicus*) alleles Spsu*01–18); XM_005338933 (thirteen‐lined ground squirrel (*Ictidomys tridecemlineatus*) allele Ictr*01); M97616 (Abert's squirrel (*Sciurus aberti*) allele Scab*01); NM_002124 (Human (*Homo sapiens*) allele HLA*01).

The colonies that we sampled generally exhibited a low level of nucleotide diversity at the *DRB1* locus, particularly at four highly similar alleles (01, 03, 05, and 06). The uncorrected nucleotide distance of these four alleles ranged from 0.015 to 0.055, and together, they reach a very high combined frequency (80–92%) across all population samples. Most of the nucleotide diversity in the *C. gunnisoni DRB1* locus is accounted for in the two remaining alleles, 02 and 04 (uncorrected distance 0.13–0.24). These two alleles appear to be trans‐species polymorphisms (Klein 1987; Takahata and Nei 1990) due to their close similarity to the *DRB1* alleles of black‐tailed prairie dogs (KR338364–KR338368) and other ground squirrels (Fig. 3). The five black‐tailed prairie dogs from Montana yielded a similar pattern of nucleotide diversity to Gunnison's prairie dog, with three alleles (MhcCylu‐DRB*01, 03, and 05) showing a close relationship (uncorrected distance = 0.011–0.027) and two additional alleles (MhcCylu‐DRB*02 and 04) contributing the majority of the observed nucleotide diversity (uncorrected distance = 0.10–0.22).

We found no evidence of positive selection acting on the *DRB1* locus during the evolutionary history of both prairie dog species. A slightly higher rate of nonsynonymous substitutions to synonymous substitutions (*d*_N_/*d*_S_ = 1.17) was observed for antigen‐binding sites (ABS) in *C. ludovicianus* only. *Z*‐tests of positive selection were nonsignificant for both species, whether considering all positions or only ABS sites (*P *>* *0.05 for all tests). In codon‐based tests (PAML), models of neutrality (the null hypothesis) were not rejected in favor of positive selection models for *C. gunnisoni*. The model incorporating positive selection fit our *C. ludovicianus* data better than models that did not incorporate this factor based on AIC values; however, the likelihood ratio test was not significant at *P *=* *0.05 (LR value = 5.812; Appendix 5). When comparing all five codon‐based models for *C. ludovicianus*, scores are similar for model 2a (the model assuming that positive selection acts on a subset of sites) and model 8 (all parameters of model 2a plus variation in *ω* is allowed that follows a beta‐distributed pattern of substitution rates). However, model 2a has fewer parameters and is therefore the most parsimonious model that explains our data.

Of the three commonly used measures of linkage disequilibrium employed by the program PERMUTE (*r* ^2^, D', and G4), only the *r* ^2^ estimate was consistent with recombination in *C. gunnisoni* (*P *=* *0.017). All three measures were nonsignificant in *C. ludovicianus*.

### Genetic variation at neutral versus MHC loci

Using 12 microsatellite markers, we observed moderate levels of neutral genetic diversity within the three prairie dog colonies, regardless of whether plague had affected the colonies or not (Appendix 6). *H*_O_ (range 0.51–0.57) and *H*_E_ (range 0.50–0.59) reached similar levels at SE, ES, and both samples of AV. All markers were in Hardy–Weinberg equilibrium in the four population samples, with the exception of marker IGS‐1 in the SE population (Appendix 6; *P *=* *0.02). Direct comparisons of *H*o (excluding the AV2006 subset) were not significantly different (Wilcoxon rank sum W = 88.1; *P *>* *0.1). The total number of alleles per locus was low (range 2–6), and no difference was observed in the mean number of alleles per population sample (3.4–4.1) (mean Wilcoxon rank sum W = 96.2; *P *>* *0.1; Appendix 6). These low‐to‐moderate diversity measures may indicate that all populations have experienced fairly similar demographic histories, regardless of whether plague was present or absent in recent decades.

The level of population structure (*F*_ST_) estimated by the microsatellite markers (Table 1) was low‐to‐moderate and did not seem to associate with the presence of plague. The mean *F*_ST_ (excluding the AV2006 subset) was 0.098 (95% CI: 0.035–0.149). No evidence of differentiation over time (*F*_ST_ = 0) was observed between the two AV population samples from 2006 and 2009 (AV2006 vs. AV_C_ in Table 1). Genetic differentiation was generally highest in pairwise comparisons that involved ES_C_ (*F*_ST_ = 0.09–0.12), which is expected given its greater geographic distance (~66 km) from the SE and AV colonies. The SE colony is <6 km distant from AV at its closest border, but was significantly differentiated from both the AV2006 and AV_C_ samples (*F*_ST_ = 0.05 and 0.08).

**Table 1:** Population structure estimates (*F*_ST_) for all pairwise population comparisons using microsatellite loci (top panel below diagonal) and the MHC *DRB1* locus (bottom panel below diagonal). Asterisks denote *P*‐value significance levels (*0.05, **0.005, ***0.0005, ns is not significant). The top panel also includes 95% confidence intervals for microsatellite *F*_ST_ estimates above the diagonal. Population samples include Aubrey Valley (AV 2006 and AV_C_); Seligman 2006 (SE); and Espee Ranch (ES_C_). Global estimates do not include the AV 2006 population sample because this would cause a downward bias from using two AV samples. The “_C_” subscript denotes AV_C_ and ES_C_ samples collected in 2009 that were used in a previous laboratory plague challenge experiment (Busch et al. 2013)

	AV 2006	SE 2006	ES_C_	AV_C_
MSAT *F*_ST_ comparisons (with 95% CI above diagonal)				
AV 2006	–	0.003–0.122	0.020–0.183	−0.012–0.022
SE 2006	0.054***	–	0.036–0.148	0.031–0.146
ES_C_	0.093***	0.091***	–	0.039–0.226
AV_C_	0.002 (ns)	0.080***	0.123***	–
Global	0.098 (0.035–0.149)			
*DRB1 F*_ST_ comparisons (below diagonal)				
AV 2006	–			
SE 2006	0.071**	–		
ES_C_	−0.005 (ns)	0.051**	–	
AV_C_	−0.004 (ns)	0.03*	0.018*	–
Global	0.033			

The MHC locus generally displayed lower levels of genetic differentiation (global *F*_ST_ = 0.033) compared to neutral microsatellite markers (Table 1). The colonies known to suffer from plague outbreaks (SE and ES_C_) displayed about half the level of differentiation at the *DRB1* locus (*F*_ST_ = 0.051, *P *<* *0.05) compared to microsatellites (*F*_ST_ = 0.091, *P *<* *0.0002). Interestingly, colonies AV_C_ and ES_C_ displayed very low differentiation at *DRB1* (*F*_ST_ = 0.018, *P *<* *0.05), which was significantly less than the 95% CI of the microsatellite loci for these two colonies (*F*_ST_ = 0.12; CI = 0.03–0.22). The relatively narrow range of *F*_ST_ variation at *DRB1* compared to microsatellite loci could be an indication of past selective pressure acting on the MHC. However, these results do not suggest a simple correlation with plague presence/absence in these prairie dog colonies.

**Figure 4:**
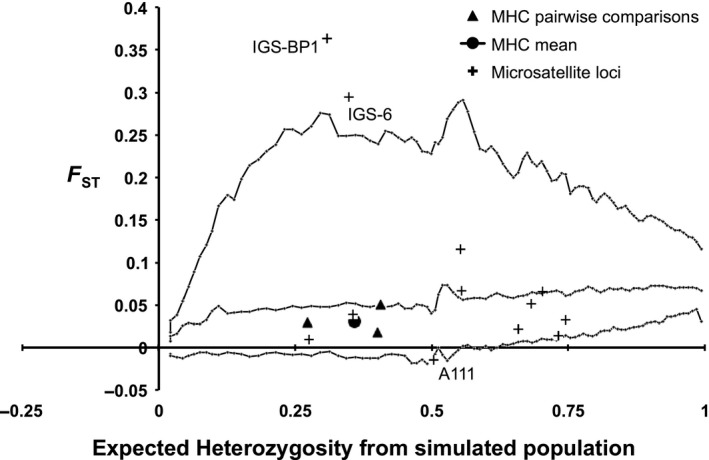
Plot of expected heterozygosity (*H*_E_) versus *F*_ST_ that tests whether the *Cynomys gunnisoni* MHC *DRB1* locus conforms to the level of genetic structure expected for neutral loci. In this analysis, the observed *F*_ST_ values from the MHC locus are directly compared to expected *F*_ST_ values from microsatellites generated with four simulated populations in fdist2 (middle line). The top and bottom lines define the 95% confidence intervals (CIs) of the simulated distribution of *F*_ST_ relative to expected heterozygosity (*H*_E_) for 12 presumably neutral microsatellite loci. *F*_ST_ values outside of the 95% CIs for neutral loci are suggestive of a locus under selection pressure. Three potentially non‐neutral loci are individually labeled (IGS‐BP1, IGS‐6, and A111). Only three of the four *Cynomys gunnisoni* populations were included in the MHC pairwise comparisons because AV was sampled twice (AV2006 and AV2009). We therefore excluded AV2006 to avoid introducing bias into this analysis. Triangles represent pairwise MHC *F*_ST_ values; the circle represents the global MHC *F*_ST_ value; crosses represent pairwise microsatellite *F*_ST_ values.

We also used a simulation technique in fdist2 to compare observed versus expected *F*_ST_ values from neutral microsatellites against the *DRB1* locus (Fig. 4). As the *DRB1* locus is subject to selection pressures (e.g., plague or other disease), we would expect to see *F*_ST_ values that are closer to zero than the neutral microsatellite loci. Although the observed *F*_ST_ estimates for the *MhcCygu‐DRB1* locus were low, they all fell within the lower 95% CI of the simulated fdist2 distributions estimated from microsatellite markers (Fig. 4). The relatively narrow range of *F*_ST_ values at *DRB1* compared to the FDIST2 simulation may suggest that past selection has acted on the MHC, but the selective signature seems to be partially masked by demography. The outlier analysis identified three microsatellite markers that are potentially non‐neutral loci: IGS‐BP1, IGS‐6, and A111 (labeled individually in Fig. 4). We therefore reran the FSTAT analysis after removing these three loci and still recovered a global *F*_ST_ estimate (*F*_ST_ = 0.065; CI = 0.04–0.09) that was significantly greater than the *DRB1* locus (0.033). It is important to note that the fdist2 result shown in Figure 4 is conservative because the simulation yielded broader 95% CIs than the FSTAT analysis.

In bottleneck tests, only the ES colony showed strong evidence of a recent population reduction (Appendix 8). Simulations under the IAM led to expected heterozygosity (*H*_E_) excess in 11 of 12 markers at ES (*P *=* *0.002). The SMM model yielded nine markers with *H*_E_ excess at ES (*P *=* *0.09). The only other colony with a bottleneck signature was SE, with 11 of 12 markers showing *H*_E_ excess under the IAM (*P *=* *0.034) but not under the SMM (*P *=* *0.733). It is worth noting that the IAM is a less conservative model and has been known to identify heterozygosity excess in nonbottlenecked populations (Luikart and Cornuet 1998). The ratio of markers with *H*_E_ deficiency versus excess was much more balanced at AV in both sample years, which is consistent with the demographic stability observed at AV in recent decades. In all colonies, the frequency of rare alleles matched that of a normal L‐shaped distribution. Hence, no bottleneck signal was observed using this method.

### Survival analyses

**Figure 5:**
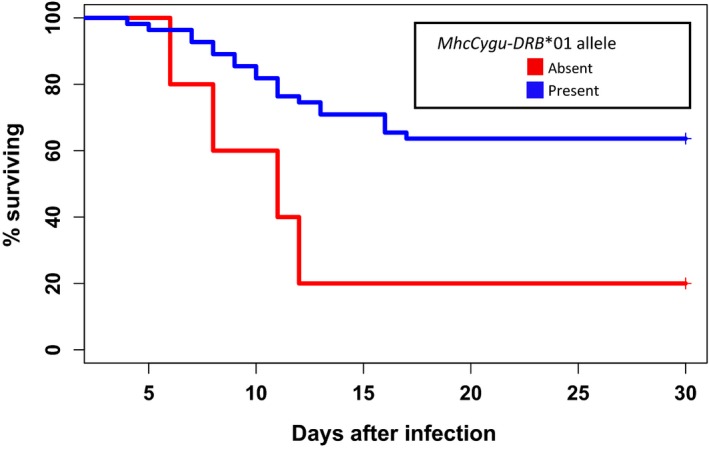
Kaplan–Meier survival curves showing that *Cynomys gunnisoni* individuals possessing at least one Mhc*Cygu*‐DRB*01 allele survived significantly longer after challenge with a single injection of *Yersinia pestis* strain CO92 compared to individuals not possessing this allele (*χ* ^2^ = 5.8, df = 1, *P *=* *0.02). Plague challenge survivorship data were collected in a previous study (Busch et al. 2013).

Comparison of Kaplan–Meier survival curves (Fig. 5) among *C. gunnisoni* subjected to *Y. pestis* challenge showed that survival rates were significantly higher in individuals that possessed at least one copy of *MhcCygu*‐DRB1*01 compared to individuals that did not possess this allele (60% vs. 20%) (*χ* ^2^ = 5.8, df = 1, *P *=* *0.02). An important caveat to this result is that the sample sizes were very unbalanced: 55 prairie dogs from ES_C_ and AV_C_ had at least one copy of allele 01, but only five individuals did not carry it. MHC heterozygosity at the *DRB1* locus was not a significant factor in determining survival during plague challenge (*χ* ^2^ = 0.1, df = 1, *P *=* *0.79), and of the 34 prairie dogs that were homozygous for allele 01, 19 survived and 15 died (Appendix 1). In contrast, every prairie dog carrying a copy of allele 04 (*n *=* *3) died when challenged with plague, including a 0104 heterozygote.

## Discussion

*Yersinia pestis* is endemic in western North America and a significant pathogen of native rodents and associated wildlife, such as black‐footed ferrets. The four species of prairie dogs in the United States have experienced recurring selection pressure from plague since the 1930s, with mortality rates approaching 100% at affected colonies (Cully et al. 1997; Cully and Williams 2001; Hoogland et al. 2004). It is possible that survival to plague is due to a genetic mechanism (as opposed to an antibody‐based mechanism) and, if so, the colonies that repopulate from a handful of resistant individuals may show improved survival during subsequent plague outbreaks. Two recent experimental challenge studies in *C. gunnisoni* and *C. ludovicianus* revealed that 50–70% of prairie dogs from plague‐endemic areas can survive a single injection of the highly virulent *Y. pestis* strain CO92 (Rocke et al. 2012; Busch et al. 2013). Survival was not correlated with the initial delivery dosage of *Y. pestis* in either prairie dog species because some animals succumbed to the lowest dose (50 bacteria) while others survived the highest dose (50,000 bacteria). This type of variation is necessary for developing an evolutionary response to a highly virulent pathogen such as *Y. pestis*. Here, we discuss the association of MHC alleles in prairie dogs experimentally challenged with plague and present the first description of MHC diversity in *C. gunnisoni*.

Variation at the *DRB1* locus in *C. gunnisoni* was lower than in most other rodent species, but similar to that observed in ground squirrels. With just six alleles, *C. gunnisoni* allelic richness is extremely low compared to mice and rats (de Bellocq et al. 2008) but about the same as seen in recently fragmented populations of alpine marmot (Kuduk et al. 2012) and spotted suslik (Biedrzycka and Radwan 2008). The low number of alleles could possibly be due to recent bottlenecks in *C. gunnisoni*, which like all prairie dog species, have experienced declines from plague, poisoning and habitat loss. Indeed, we found evidence of recent bottlenecks at the two plague sites (SE and ES), although not at AV. Fortunately, the nucleotide diversity among existing alleles in *C. gunnisoni* (0.015–0.24) and C. *ludovicianus* (0.011–0.22) remains high and is similar to that reported for other ground squirrel species (Biedrzycka and Radwan 2008; Kuduk et al. 2012; Moreno‐Cugnon et al. 2015). The co‐occurrence of highly divergent *DRB1* alleles provides evidence that balancing selection has occurred in the course of prairie dog evolution, as expected for this class II MHC locus. In contrast to the high level of nucleotide diversity at *DRB1*, levels of *H*_E_ were low in *C. gunnisoni* (0.27–0.53) compared to spotted suslik (0.63–0.89), eastern woodchuck (0.77–0.83), and alpine marmot (0.51–0.63) (Biedrzycka and Radwan 2008; Kuduk et al. 2012; Moreno‐Cugnon et al. 2015). *DRB1* heterozygosity in *C. gunnisoni* was also lower than *H*_E_ at microsatellite loci (0.50–0.59). These microsatellite *H*_E_ estimates fell in the middle range of *C. ludovicianus* (0.53–0.63) (Roach et al. 2001; Sackett et al. 2012, 2013; Castellanos‐Morales et al. 2015) and other sciurids (0.33–0.75) (Stevens et al. 1997; Reid et al. 2010; Ricanova et al. 2011; Fitak et al. 2013) and suggests that *C. gunnisoni* is maintaining about the same level of genetic diversity as these phylogenetic relatives. The level of genetic structure among the three *C. gunnisoni* populations we studied was somewhat less than regional differentiation in *C. gunnisoni zuniensis* (0.38) (Sackett et al. 2014), *C. ludovicianus* (0.54) (Sackett et al. 2012, 2013; Castellanos‐Morales et al. 2015), and *C. mexicanus* (0.17) (Castellanos‐Morales et al. 2015). As such, the level of genetic differentiation and diversity at microsatellites in our study populations seems typical for prairie dogs and other ground squirrel species.

Selection appears to have played an important role in shaping patterns of genetic structure at the MHC *DRB1* locus in *C. gunnisoni*. This has led to a reduced level of genetic differentiation (*F*_ST_) at the MHC locus compared to the level at neutral markers (Table 1). The variation in pairwise *DRB1 F*_ST_ estimates (0.018–0.051) is also much narrower than that for 12 microsatellites (Fig. 4). However, two lines of evidence suggest that demography has also imparted a detectable signal on the *DRB1* locus. First, all of the pairwise *F*_ST_ values for *DRB1* lie inside the 95% confidence interval generated by the fdist2 simulation based on microsatellite markers (Fig. 3). The same outcome has been observed in another ground squirrel, the spotted suslik (*Spermophilus suslicus*) (Biedrzycka and Radwan 2008). In both species, genetic drift appears to be more important than balancing selection in shaping MHC variation. This pattern has also been reported in other strongly bottlenecked or fragmented populations (Miller and Lambert 2004; Strand et al. 2012; Sutton et al. 2015). Second, we did not find evidence of positive selection acting on specific amino acid positions in *C. gunnisoni*, even though we did find this in *C. ludovicianus*. Genetic drift may explain the absence of this expected selective signature. Additional explanations may be that epizootic plague is not the only source of selection in these colonies and plague does not affect every prairie dog generation. Assuming a generation time of 1–2 years, *C. gunnisoni* has existed with endemic plague for a maximum of 85 generations and perhaps not enough time has elapsed to detect an evolutionary response at MHC loci. Another mechanism that can explain variation in *F*_ST_ values at the *DRB1* locus is that selection pressures on the MHC can fluctuate over time and space (Hill 1991; Hedrick 2002). In this case, determining the relative influence of plague may require sampling at a broader geographic scale. It is known that the average *F*_ST_ among 34 rangewide samples of *C. gunnisoni* subspecies *zuniensis* is 0.38 for microsatellite markers (Sackett et al. 2014), which is considerably greater than the level we observed (microsatellite *F*_ST_ 0.08–0.123 for AV‐SE‐ES). It is possible that MHC differentiation across these same 34 rangewide locations will be much lower.

In genotyping the 60 *C. gunnisoni* that were used in the previous experimental plague challenge (Busch et al. 2013), we found evidence for a potential MHC‐based survival advantage in individuals that carried at least one copy of allele MhcCygu‐DRB1*01. Despite a large number of challenge studies on laboratory rodents, only a handful of plague challenge studies have been conducted using wild rodents besides prairie dogs (Quan and Kartman 1962; Rust et al. 1972; Quan et al. 1985; Thomas et al. 1988, 1989; Tollenaere et al. 2010b). Although the presence of the 01 allele does not guarantee survival during a plague infection, the absence of this allele appears to be disadvantageous (60% vs. 20% survival). Individuals carrying allele 04 seemed particularly ill‐suited for fighting plague, as the three prairie dogs with this allele all succumbed to the infection, including a 0104 heterozygote. We recognize the potential problem of imbalanced sample size for these allelic comparisons, as 55 prairie dogs had at least one copy of the 01 allele but only five individuals lacked this allele. The pattern that we observed might suggest the common MHC allele is an important factor for prairie dog survival during plague infection, but it is likely not the only factor. It may also be possible that plague survival is influenced by a closely linked gene rather than this MHC class II locus. The six qPCR assays that we developed will facilitate rapid MHC genotyping in future studies of prairie dogs and plague.

We found evidence of trans‐species polymorphisms for two allelic lineages (02 and 04) that shared high sequence similarity between *C. gunnisoni* and *C. ludovicianus* (Fig. 3). These two species do not hybridize and are members of different subgenera that last shared a common ancestor about five million years ago (Goodwin 1995; Herron et al. 2004). Alleles 02 and 04 have been maintained over an evolutionary time frame by *C. gunnisoni* and *C. ludovicianus* with only a small number of species‐specific changes (Appendix 3). Therefore, we predict that very similar alleles will also be found in *C. parvidens* and *C. leucurus*. The maintenance of ancient polymorphisms through speciation is frequently observed in MHC loci (Klein 1987; Figueroa et al. 1988; Sena et al. 2003; Califf et al. 2013) and may be due to balancing selection over extended evolutionary timescales (Takahata and Nei 1990) or convergent evolution from similar pathogen pressures (Edwards and Hedrick 1998; Yeager and Hughes 1999). Some of the prairie dog alleles share past ancestry with major allele lineages found in the spotted suslik (*Spermophilus suslicus*), an Asian ground squirrel that naturally occurs in areas with native plague. Ancient polymorphisms shared by ground squirrels species would probably have arisen before the divergence of *Y. pestis* from its ancestor (*Y. pseudotuberculosis*) in the past 6000 years (Cui et al. 2013; Rasmussen et al. 2015). In a broad sense, this means that these MHC alleles could still be experiencing selection by this recently evolved pathogen. It remains to be seen whether all prairie dog species share MHC allele lineages that may provide a survival advantage during *Y. pestis* infection.

Most of what is currently known about the genetic basis of plague resistance is based on evidence obtained from laboratory experiments on model organisms. The existing studies suggest that plague resistance is a polygenic trait, probably because *Y. pestis* attacks the immune system with a complex array of virulence mechanisms. Our study is generally consistent with this idea, because the class II MHC locus is clearly not the only factor determining, which prairie dogs will survive plague. In the prairie dog challenge experiments, very few individuals of either species produced a strong antibody response, indicating that other immune mechanisms (e.g., early inflammation or innate pathways) may be more critical for surviving plague infection (Rocke et al. 2012; Busch et al. 2013). In laboratory mice, at least four plague resistance loci (*prls*) contribute to high resistance levels in *Mus spretus* (Blanchet et al. 2011; Chevallier et al. 2013) and another *prl* maps to the MHC region of *M. musculus* line BALB/cJ (Turner et al. 2008). Resistance in *M. musculus* line SEG is associated with rapid induction of early innate immune responses that occur in the first 2–3 days of infection (Demeure et al. 2011). The MHC class II apparatus is known to present the epitopes from several *Y. pestis* proteins (Parent et al. 2005; Shim et al. 2006), including the F1 and LcrV antigens that are used as a plague vaccine for prairie dogs (Mencher et al. 2004; Rocke et al. 2008, 2010). However, *Y. pestis* can use an effector protein (*Yop* M) to suppress MHC class II antigen presentation (Soundararajan et al. 2011; Bosio et al. 2012) that likely serves as a mechanism to interrupt the activation of T cells. The results of our challenge study raise the possibility that some class II alleles might be more easily suppressed with this type of mechanism than others. It could also be that certain prairie dog alleles are simply less effective at binding and presenting *Y. pestis* antigens. The application of genomewide scans to find new plague resistance markers (Tollenaere et al. 2010a, 2013), including those within the MHC (Tollenaere et al. 2012), will hopefully lead to a better understanding of the complex immune responses that mammals employ during exposure to plague.

## Conclusions

Characterizing genes integral to the immune system is an important first step in identifying possible mechanisms of resistance to diseases in nonmodel species like prairie dogs. This is the first published report of an expressed MHC class II *DRB1* locus in any prairie dog species. The small number of alleles at this *DRB1* locus all share similarity to other ground squirrels, including *C. ludovicianus* and the spotted suslik (*S. suslicus*). We found weak evidence for positive selection acting on the *DRB1* locus in the black‐tailed prairie dog, but not in Gunnison's prairie dog, which may be a result of recent population reductions or the short time frame during which plague has been acting as a selective force. Plague is probably not the only source of selective pressure in this system, and other diseases (e.g., *Francisella tularensis*) or parasites may play an important role in shaping variation at immune system loci in prairie dogs (Zeidner et al. 2004). In addition to disease, two other important factors have contributed to major declines in all prairie dog species in the last 100 years: habitat conversion and poisoning campaigns (Roemer and Forrest 1996; Wagner et al. 2006). In combination, these factors may have contributed to reductions in the genomic diversity found in *C. gunnisoni* at both neutral loci and genes under selection. We are currently investigating whether greater microsatellite and MHC diversity existed prior to the introduction of plague to the western United States by genotyping *C. gunnisoni* specimens collected prior to 1930. These studies will contribute to an understanding of the development of plague resistance in prairie dogs and other rodents, and help elucidate the influence of diseases in shaping natural genetic variation in wild populations.

## Conflict of Interest

The authors declare no conflict of interest.

## Data Archiving

Sequence data have been submitted to GenBank under accession numbers GenBank accessions KU695893–KU695896 and KR338362–KR338368.

## Appendix 1

Sample data for 94 *Cynomys gunnisoni* individuals collected from three locations in Arizona: Aubrey Valley (AV), Espee Ranch (ES), and Seligman (SE). The subscripted “_C_” for AV_C_ and ES_C_ denotes the subset of samples collected in 2009 that were used in a previous experimental challenge with *Y. pestis* (Busch et al. 2013). Additional data for these samples includes sex (male [M], female [F], or unknown [U]); age (adult [A], juvenile [J] or unknown [U]); capture weight; the infectious dose of *Y. pestis* strain CO92 delivered to each prairie dog in colony forming units (cfu); fate (survived [SURV] or died [DIED]); days post infection (DPI) until time of euthanization for moribund animals (all survivors were euthanized after 30 days); and *DRB1* alleles for each individual.

**Table:**

Sample	Pop	Collection date	Zone 12N UTM_N	Zone 12N UTM_E	Sex	Age	Capture wt. (g)	Inf. dose (cfu)	Fate	DPI	*DRB1* allele 1	*DRB1* allele 2
AV01^V^	AV	24AUG2005	3916926	320515	M	A	–	–	–	–	02	03
AV02	AV	24AUG2005	3917126	320555	F	A	–	–	–	–	02	06^m^
AV03^V^	AV	24AUG2005	3917823	320968	M	A	–	–	–	–	01	02
AV04	AV	24AUG2005	3918959	320552	M	A	–	–	–	–	01	01
AV05	AV	24AUG2005	3918994	320603	M	A	–	–	–	–	01	01
AV06	AV	24AUG2005	3919256	320725	F	A	–	–	–	–	01	01
AV09	AV	26AUG2006	3921136	314603	U	U	–	–	–	–	01	02
AV10	AV	26AUG2006	3922121	313441	F	U	–	–	–	–	01	01
AV11	AV	26AUG2006	3922420	313017	U	U	–	–	–	–	01	01
AV12	AV	26AUG2006	3922520	313161	F	U	–	–	–	–	01	03
AV13	AV	26AUG2006	3922748	312612	M	U	–	–	–	–	01	03
AV14	AV	26AUG2006	3927834	310982	M	U	–	–	–	–	01	01
AV15	AV	26AUG2006	3926003	308507	M	U	–	–	–	–	01	02
AV16	AV	26AUG2006	3922808	312411	M	U	–	–	–	–	01	03
AV17	AV	26AUG2006	3922615	312647	F	U	–	–	–	–	01	01
AV18	AV	26AUG2006	3922593	312522	U	U	–	–	–	–	01	01
SE07	SE	09SEP2006	3909725	327090	U	U	–	–	–	–	01	01
SE08	SE	09SEP2006	3908249	325340	F	U	–	–	–	–	01	06^m^
SE19	SE	09SEP2006	3909656	327066	M	A	–	–	–	–	01	01
SE20	SE	09SEP2006	3907874	325037	M	U	–	–	–	–	01	01
SE21	SE	09SEP2006	3907805	325694	F	U	–	–	–	–	01	01
SE22	SE	09SEP2006	3907669	325116	M	U	–	–	–	–	01	01
SE23	SE	09SEP2006	3907338	325471	M	U	–	–	–	–	01	01
SE24	SE	09SEP2006	3906958	325301	M	U	–	–	–	–	01	01
SE25^V^	SE	09SEP2006	3907279	325606	M	U	–	–	–	–	01	04
SE26	SE	09SEP2006	3907775	325014	M	U	–	–	–	–	01	06^m^
SE27	SE	09SEP2006	3911917	327397	M	U	–	–	–	–	01	01
SE28	SE	09SEP2006	3907673	330195	M	U	–	–	–	–	01	01
SE29	SE	09SEP2006	3913203	332392	M	U	–	–	–	–	01	01
SE30	SE	09SEP2006	3914578	332705	M	U	–	–	–	–	01	01
SE31^V^	SE	09SEP2006	3913326	327153	M	U	–	–	–	–	01	04
SE32	SE	09SEP2006	3913218	327220	F	U	–	–	–	–	01	01
SE33	SE	09SEP2006	3912854	327322	F	U	–	–	–	–	01	01
SE34	SE	09SEP2006	3911973	327342	F	U	–	–	–	–	01	04
60‐28	ES_C_	01SEP2009	3961380	356869	F	J	500	50000	SURV	30	01	01
61‐30	ES_C_	01SEP2009	3961020	357068	F	A	850	50000	DIED	9	01	01
63‐29^V^	ES_C_	01SEP2009	3961430	356880	M	J	750	50	DIED	12	02	02
64‐19	ES_C_	31AUG2009	3967379	360787	M	J	700	50000	SURV	30	01	03
65‐20	ES_C_	01SEP2009	3967386	360711	M	J	550	50	DIED	8	01	02
66‐23	ES_C_	01SEP2009	3967438	360489	M	A	1100	50000	SURV	30	01	01
67‐24	ES_C_	01SEP2009	3967226	360624	M	A	1000	50	SURV	30	01	01
68‐33	ES_C_	01SEP2009	3961114	356884	M	A	1175	5000	DIED	10	01	01
69‐04	ES_C_	25AUG2009	3961396	356886	F	J	525	5000	SURV	30	01	01
70‐22	ES_C_	01SEP2009	3967386	360723	F	J	475	50	SURV	30	01	05^m^
71‐17^V^	ES_C_	29AUG2009	3961555	357242	M	J	700	50000	DIED	11	02	04
72‐12	ES_C_	29AUG2009	3967200	360621	F	J	575	5000	DIED	16	01	01
73‐31^V^	ES_C_	01SEP2009	3961575	357274	F	A	675	5000	DIED	8	04	06^m^
74‐35	ES_C_	01SEP2009	3967193	360751	M	J	500	5000	DIED	4	01	03
75‐32	ES_C_	01SEP2009	3961076	357066	F	J	625	5000	SURV	30	01	01
76‐21	ES_C_	01SEP2009	3967365	360690	F	J	500	50000	DIED	16	01	01
77‐01	ES_C_	29AUG2009	3967194	360872	M	J	750	5000	DIED	13	01	04
78‐08	ES_C_	25AUG2009	3967393	360730	M	A	850	50000	DIED	16	01	01
79‐16	ES_C_	29AUG2009	3967423	360681	M	J	650	50000	DIED	10	01	01
80‐34	ES_C_	01SEP2009	3961086	356990	F	A	675	50	SURV	30	01	06^m^
81‐05	ES_C_	25AUG2009	3961380	356869	F	J	525	50	SURV	30	01	05^m^
82‐03	ES_C_	25AUG2009	3961387	356881	M	A	925	50	DIED	12	01	01
83‐36	ES_C_	04SEP2009	3967525	360500	M	J	600	50	SURV	30	01	05^m^
85‐18	ES_C_	29AUG2009	3961083	357887	F	J	600	50	SURV	30	01	06^m^
86‐06	ES_C_	25AUG2009	3961362	356862	M	J	600	5000	SURV	30	01	02
87‐25	ES_C_	01SEP2009	3967535	360540	M	J	700	50000	SURV	30	01	01
88‐81	ES_C_	29AUG2009	3967373	360804	M	J	450	50	DIED	13	01	02
89‐02	ES_C_	25AUG2009	3961426	356866	F	A	700	5000	SURV	30	01	02
90‐07	ES_C_	25AUG2009	3961349	356873	F	A	750	50000	DIED	11	01	02
127‐14	ES_C_	29AUG2009	3967395	360730	M	J	575	5000	SURV	30	01	02
92‐49	AV_C_	15SEP2009	3928536	309811	M	J	650	50000	SURV	30	01	01
93‐56	AV_C_	15SEP2009	3922057	312544	F	A	700	50	SURV	30	01	02
94‐40	AV_C_	15SEP2009	3927905	308962	M	J	675	50	SURV	30	01	01
95‐65	AV_C_	18SEP2009	3921130	313790	M	J	625	5000	SURV	30	01	01
96‐53	AV_C_	15SEP2009	3922191	312523	M	A	1000	50000	SURV	30	01	01
97‐57^V^	AV_C_	15SEP2009	3921451	313231	M	J	775	5000	DIED	6	02	02
98‐63	AV_C_	18SEP2009	3921070	313751	M	J	750	50000	SURV	30	01	03
99‐69	AV_C_	18SEP2009	3921837	313308	M	A	800	5000	DIED	11	01	01
100‐46	AV_C_	15SEP2009	3927956	309420	F	A	775	5000	SURV	30	01	01
101‐54	AV_C_	15SEP2009	3922177	312600	M	J	725	5000	SURV	30	01	01
102‐62	AV_C_	18SEP2009	3927972	309418	M	A	1150	50	SURV	30	01	01
103‐11	AV_C_	28AUG2009	3922013	313157	M	J	750	50000	DIED	7	01	01
104‐43	AV_C_	15SEP2009	3928034	309467	F	J	525	5000	SURV	30	01	01
105‐55^V^	AV_C_	15SEP2009	3922100	312478	M	J	750	50	SURV	30	02	03
106‐09	AV_C_	28AUG2009	3922043	313167	F	A	700	50000	DIED	9	01	01
107‐67	AV_C_	18SEP2009	3921785	313179	F	J	550	50000	SURV	30	01	02
108‐66	AV_C_	18SEP2009	3921236	313762	F	A	775	5000	DIED	11	01	01
110‐48	AV_C_	15SEP2009	3928476	309863	F	J	550	5000	DIED	8	01	01
112‐45	AV_C_	15SEP2009	3928004	309439	M	J	600	50000	SURV	30	01	01
114‐51	AV_C_	15SEP2009	3922434	312012	M	A	875	50	SURV	30	01	01
115‐44	AV_C_	15SEP2009	3927901	309393	F	J	500	50	SURV	30	01	01
116‐61	AV_C_	18SEP2009	3928026	309279	F	A	650	50000	DIED	5	01	01
117‐41^V^	AV_C_	15SEP2009	3927918	308938	M	J	725	5000	SURV	30	01	03
118‐68	AV_C_	18SEP2009	3921579	313264	M	A	850	50000	SURV	30	01	02
119‐47	AV_C_	15SEP2009	3928460	309703	M	J	750	50	SURV	30	01	02
120‐58	AV_C_	15SEP2009	3921514	313260	M	J	750	50	SURV	30	01	02
121‐10	AV_C_	28AUG2009	3922876	312444	F	J	625	50	DIED	7	01	01
122‐50	AV_C_	15SEP2009	3922376	311977	F	J	600	50	SURV	30	01	01
123‐42	AV_C_	15SEP2009	3928066	309474	F	J	625	50000	SURV	30	01	01
124‐52	AV_C_	15SEP2009	3922546	312087	M	A	1050	5000	DIED	17	01	01

^m^“Missing allele” that was not observed in sequences generated from the Sanger and 454 platforms.

^V^“Validation individual” used to confirm expression of the *DRB1* gene using mRNA from spleen tissue.

## Appendix 2

Primers used for sequencing and running quantitative PCR assays on *Cynomys gunnisoni DRB1* alleles. *T*_a_ is the annealing temperature in PCR; Melt temp is the temperature at which double‐stranded PCR amplicons reach their greatest dissociation rate on an AB7500‐FAST instrument. In primer names, Anc = putative ancestral state of mutation being assayed; Der = putative derived state of mutation being assayed.

**Table:**

Name	Sequence (5′**–**3′)	Target allele	*T*_a_ (°C)	Size (bp)	Melt temp (°C)	Comments
**PCR and sequencing primers**						
GH46F	CCGGATCCTTCGTGTCCCCACAGCACG	All	65	271	–	Erlich and Bugawan 1990
GH50R	CTCCCCAACCCCGTAGTTGTGTCTGCA	All	65	271	–	Erlich and Bugawan 1990
*CyguDRB1*_exon2_R1	CTCTCCTCTCCACAGTGAAGCTCTCA	All (exon 2) except *Cygu‐DRB1**03	65	293		New reverse primer specific to *C. gunnisoni*.
*CyguDRB1*_exon2_R2	CTCTCCGCTCCACAGCGAAGCTCTCA	*Cygu‐DRB1**03	65	293	–	New reverse primer specific to *C. gunnisoni*.
*CyguDRB1*exon3‐R2	AGACCAGGAGGTTGTGGTGCTG	All (exon 3)	65	334	–	For cDNA PCR and sequencing.
454 adapter‐A	cgtatcgcctccctcgcgccatcagNNNNNNNNNN	–	64	306	–	Tail for GH46F (with 10 bp indices).
454 adapter‐B	ctatgcgccttgccagcccgctcag	–	64	296	–	Tail for reverse primer (e.g., GH50R or *CyguDRB1*_exon2_R1 and R2).
**Assays 1–4: Noncompetitive PCRs for all known *CyguDRB1* alleles**						
PD1*DRB*_F	CGTTTCCTGGAGCAAGTTTCACATG	All	67	96	–	This forward primer can amplify all six alleles.
PD1*DRB*_R	CGAACTCCTCTCGGTTGTGG	*Cygu‐DRB1**01 *Cygu‐DRB1**05 *Cygu‐DRB1**06	67	96	80–81	This reverse primer amplifies three highly similar alleles (01, 05, and 06).
PD2*DRB*_F2	GGAGCGGATACGGTTCCTAC	*Cygu‐DRB1**02	67	48	73.5–74.5	
PD2*DRB*_R	GACCTCCTCCCGGTTATAGAT	*Cygu‐DRB1**02	67	48	73.5–74.5	
PD3*DRB*_F2	TTTCCTGGAGCAAGGTTCACA	*Cygu‐DRB1**03	67	102	80.75–81.75	
PD3*DRB*_R2	AAAGCGCACGTACTCCTCCA	*Cygu‐DRB1**03	67	102	80.75–81.75	
PD4*DRB*_F2	GTTTCCTGGAGCAAGGTTTCAG	*Cygu‐DRB1**04	67	183	86–87	
PD4*DRB*_R2	GCGTCCTTCTGGCTGTTGA	*Cygu‐DRB1**04	67	183	86–87	
**Assay 5: Noncompetitive PCRs to resolve allele 01**						
PD5*DRB*_F	CGTTTCCTGGAGCAAGTTTCACATG	All	65		–	Same sequence as primer PD1*DRB*_F above.
PD5*DRB*_R2_Anc	CCTCTCCACAGTGAAGCTCTCACCA	*Cygu‐DRB1**01 (poor amp for 05, 06)	67	264	87–88	PCR with this reverse primer will amplify three highly similar alleles (01, 05, and 06). However, the *C*_t_ value for allele 01 will occur 8–10 cycles earlier compared to a PCR using the alternate reverse primer (PD5*DRB*_R2_Der).
PD5*DRB*_R2_Der	TCTCCTCTCCACAGTGAAGCTCTCAAAC	*Cygu‐DRB1**05 *Cygu‐DRB1**06 (poor amp for 01)	67	267	87–88	PCR with this reverse primer will amplify three highly similar alleles (01, 05, and 06). However, the *C*_t_ value for alleles 05 or 06 will occur 8–10 cycles earlier compared to a PCR using the alternate reverse primer (PD5*DRB*_R2_Anc).
**Assay 6: Competitive PCR to resolve allele 05 versus 06**						
PD6*DRB*_164G_F_Anc	AGTACCGCGCGGTGAGAG	*Cygu‐DRB1**01 *Cygu‐DRB1**05	56	33	75.5–76.5 and 84–85	Double peaks observed in dissociation curve.
PD6*DRB*_164A_F_Der	gcccgcccgcccgcccAGTACCGCGCGGTGAGTA	*Cygu‐DRB1**06	56	49	82–83	
PD6*DRB*_R_Cons	CCGGCCGCCCCAGCT	All alleles (two SNPs in D allele)	56		–	Conserved reverse primer.

### Methods for qPCR assays

To provide rapid and accurate identification of the known *DRB1* alleles in western Arizona populations of *C. gunnisoni*, we designed six quantitative PCR (qPCR) assays. Our genotyping process requires three rounds of PCR. First, assays 1–4 are run to detect all six known alleles (Appendix 3). Second, the subset of individuals that appear to have at least one *Cygu*‐*DRB1**01 allele are tested with assay 5 to distinguish allele 01 from 05 or 06. Third, a competitive PCR with two forward primers (assay 6) is used to unambiguously resolve alleles 05 and 06.

The first four assays (Appendix 2) use noncompetitive PCRs to detect all six *Cygu*‐*DRB1* alleles using the dissociation curve (melting temperature) feature of AB7500 or AB7900 instruments (Applied Biosystems, Foster City, CA). PCRs were carried out in 10 *μ*L volumes containing the following reagents (given in final concentrations): 20 ng gDNA template, 1X SYBR^®^ Green Master Mix (Invitrogen, Carlsbad, CA), and 0.15 *μ*M of each forward and reverse primer pair. PCRs were thermocycled on an AB7500 (Applied Biosystems, Foster City, CA) under the following conditions: 95°C for 10 min to release the polymerase antibody, followed by 35 cycles of 95°C for 15 sec, 67°C for 60 sec, 72°C for 30 sec. Afterward, a dissociation curve was generated for each sample. Alleles *Cygu*‐*DRB1**01, 05, and 06 dissociate at 80–81°C, allele 02 dissociates at 73.5–74.5°C, allele 03 dissociates at 80.75–81.75°C, and allele 04 dissociates at 86–87°C. We included positive control templates in all runs. After this first PCR, alleles *Cygu*‐*DRB1**02, 03, and 04 required no further validation. However, all genotypes that contained alleles 01, 05, or 06 required two additional assays because of the high sequence similarity shared by these three alleles.

The fifth assay requires two noncompetitive PCRs that use the same forward primer (PD5*DRB*_F) but different reverse primers (PD5*DRB*_R2_Anc or PD5*DRB*_R2_Der) to distinguish allele *Cygu*‐*DRB1**01 from 05 and 06 (Appendix 2). PCRs were carried out in 10 *μ*L volumes containing the following reagents (given in final concentrations): 20 ng gDNA template, 1X SYBR^®^ Green Master Mix, 0.15 *μ*M of each primer (two forwards and one reverse). Reactions were thermocycled on an AB7500 under the following conditions: 95°C for 10 min to release the polymerase antibody, followed by 35 cycles of 95°C for 15 sec, 67°C for 60 sec, 72°C for 30 sec. We ran gDNA controls in all runs. Afterward, a dissociation curve was generated for each sample. Due to nearly identical fragment sizes (264 bp vs. 267 bp), all three alleles (*Cygu*‐*DRB1**01, 05, and 06) dissociate at 87**–**88°C. Genotypes were therefore determined by comparing the threshold cycle (*C*_t_) of the two PCRs. The PCR with reverse primer PD5*DRB*_R2_Anc favors amplification of allele *Cygu*‐*DRB1**01 and yields a *C*_t_ value approximately 8**–**10 cycles earlier than the other PCR containing reverse primer PD5*DRB*_R2_Der, which provides only poor amplification for allele 01. The opposite situation produces a comparable result: a PCR with reverse primer PD5*DRB*_R2_Der will favor alleles 05 and 06 and yield a *C*_t_ value approximately 8**–**10 cycles earlier than a PCR primed by reverse primer PD5*DRB*_R2_Anc. For heterozygous individuals that carry one copy of the 01 allele and one copy of either 05 or 06, both PCRs amplify robustly and produce amplification plots within one *C*_t_ of each other. At this point, alleles that genotyped as *Cygu*‐*DRB1**01 required no further validation. However, any individuals that carried at least one 05 or 06 allele required a third step to determine the state of a single nucleotide polymorphism (SNP) at base 164 of exon 2.

Assay 6 (Appendix 2) was a competitive PCR that used a conserved reverse primer (PD6*DRB*_R_Cons) in combination with two forward primers (PD6*DRB*_164G_F_Anc and PD6*DRB*_164A_F_Der) designed to identify a G nucleotide (presumed to be the ancestral state in alleles 01 and 05) or an A nucleotide (putatively, the derived state from allele 06) at base 164 of exon 2. All three primers were used in each PCR; reactions were carried out in 10 *μ*L volumes containing the following reagents (given in final concentrations): 20 ng of gDNA template, 1X SYBR^®^ Green Master Mix, 0.15 *μ*M of each forward primer and 0.30 *μ*M of the reverse primer. Quantitative PCRs were thermocycled on an AB7500 under the following conditions: 95°C for 10 min to release the polymerase antibody, followed by 35 cycles of 95°C for 15 sec, 56°C for 60 sec, 72°C for 30 sec. We included gDNA control templates in all runs. Afterward, a dissociation curve was generated for each sample. Amplicons derived from the primer PD6*DRB*_164G_F_Anc produced a melt curve with two dissociation peaks: one at 75.5**–**76.5°C and another at 84**–**85°C; this result identified allele 01 or 05. The derived product from primer PD6*DRB*_164A_F_Anc yielded a single dissociation peak at 82**–**83°C that identified allele 06. Heterozygote individuals carrying alleles 05 and 06 should produce all three of these dissociation peaks; however, both alleles are found in very low frequency and will rarely be observed together in a single individual.

### Difficult samples

For samples with low quality/quantity DNA, we added Platinum^®^ *Taq* polymerase (extra 0.04U) to each PCR to enhance the performance of all six assays. If any assay results were still ambiguous (a rare occurrence), exon 2 alleles were determined by Sanger sequencing cloned PCR products from the problem individual. To insure that all exon 2 alleles would amplify without bias, we used forward primer GH46F with two new reverse primers (*CyguDRB1*_exon2_R1 and *CyguDRB1*_exon2_R2) that are specific to *C. gunnisoni* (Appendix 2).

## Appendix 3

Nucleotide alignment of MHC class II *DRB1* sequences from two prairie dog species (*Cynomys gunnisoni* and *C. ludovicianus*). All *C. gunnisoni* sequences are derived from mRNA as a starting template, except positions 5**–**27 which were derived from DNA templates; all *C. ludovicianus* sequences are derived from DNA only. *Dots* represent identity to the top sequence (*MhcCygu‐DRB1**01); *dashes* represent missing data; *arrows* mark the start of exons 2 and 3; *shading* on reverse primer GH50R indicates six positions that are mismatched for alleles 05 and 06. Nucleotide positions are numbered according to *HLA‐DRB1* (NM_002124). All sequences have been deposited in GenBank (accession numbers KU695893–KU695896 and KR338362–KR338363 for *C. gunnisoni* and KR338364–KR338368 for *C. ludovicianus*).

## Appendix 4

Expected results from six qPCR assays used to genotype *Cynomys gunnisoni* MHC *DRB1* alleles. The following characters represent PCR outcomes: 0 = no amplification; 1 = successful amplification; Anc = ancestral state; Der = derived state; “n/a” = assay not required (depending on the outcome of assays 1**–**4).

**Table:**

	Outcome of 6 qPCR assays						Comments
*DRB1* alleles	1	2	3	4	5	6	
**By allele**						
*Cygu‐DRB1**01	1	0	0	0	Anc	Anc	Most common allele in western AZ populations
*Cygu‐DRB1**02	0	1	0	0	n/a	n/a	
*Cygu‐DRB1**03	0	0	1	0	n/a	n/a	
*Cygu‐DRB1**04	0	0	0	1	n/a	n/a	
*Cygu‐DRB1**05	1	0	0	0	Der	Anc	Very similar to allele *Cygu‐DRB1**01
*Cygu‐DRB1**06	1	0	0	0	Der	Der	Very similar to allele *Cygu‐DRB1**01
**By diploid genotype**							
0101	1	0	0	0	Anc	Anc	Most common diploid genotype
0102	1	1	0	0	Anc	Anc	
0103	1	0	1	0	Anc	Anc	
0104	1	0	0	1	Anc	Anc	
0105	1	0	0	0	Anc + Der	Anc	
0106	1	0	0	0	Anc + Der	Der	
0202	0	1	0	0	n/a	n/a	
0203	0	1	1	0	n/a	n/a	
0204	0	1	0	1	n/a	n/a	
0205	1	1	0	0	Der	Anc	Not observed
0206	1	1	0	0	Der	Der	
0303	0	0	1	0	n/a	n/a	Not observed
0304	0	0	1	1	n/a	n/a	Not observed
0305	1	0	1	0	Der	Anc	Not observed
0306	1	0	1	0	Der	Der	Not observed
0404	0	0	0	1	n/a	n/a	Not observed
0405	1	0	0	1	Der	Anc	Not observed
0406	1	0	0	1	Der	Der	
0505	1	0	0	0	Der	Anc	Not observed
0506	1	0	0	0	Der	Anc + Der	Not observed
0606	1	0	0	0	Der	Der	Not observed

## Appendix 5

Evaluation of codon‐based evolution models at exon 2 of the MHC class II *DRB1* locus in *Cynomys gunnisoni* and *C. ludovicianus*. The best model is highlighted in **bold** font for both species.

**Table:**

*Cynomys gunnisoni*			
Model†	ln *L* §	ΔAIC	Parametersφ
M0 – one *ω*	−675.361	37.50	0.290
**M1a – nearly neutral (*ω***_**0**_ ** < 1, *ω***_**1**_ ** = 1)**	**−655.611**	**best** *	**p**_**0**_ ** = 0.733, *ω***_**0**_ ** = 0.034**
M2a – positive selection (*ω*_0_ < 1, *ω*_1_ = 1, *ω*_2_ > 1)	−653.777	0.331	p_0_ = 0.805, p_2_ = 0.195,*ω*_0_ = 0.073, *ω*_2_ = 2.145
M7 – beta (*p*,* q*)	−656.058	0.892	*p* = 0.017; *q* = 0.034
M8 – beta and *ω* (*p*,* q*,* ω*s > 1)	−653.782	0.341	p_0_ = 0.806, (p_1_ = 0.194), *p* = 8.015, *q* = 99.0, *ω* = 2.155
*Cynomys ludovicianus*			
Model†	ln *L* §	ΔAIC	Parametersφ
M0 – one *ω*	−552.710	26.682	0.325
M1a – nearly neutral (*ω*_0_ < 1, *ω*_1_ = 1)	−542.278	5.818	p_0_ = 0.654, *ω*_0_ = 0.059
**M2a – positive selection (*ω***_**0**_ ** < 1, *ω***_**1**_ ** = 1, *ω***_**2**_ ** > 1)**	**−539.369**	**best** *	**p**_**0**_ ** = 0.631, p**_**2**_ ** = 0.091, *ω***_**0**_ ** = 0.089, *ω***_**2**_ ** = 13.813**
M7 – beta (*p*,* q*)	−542.612	6.487	*p* = 0.111; *q* = 0.171
M8 – beta and ω (*p*,* q*,* ω*s > 1)	−539.275	−0.187	p_0_ = 0.905, (p_1_ = 0.095), *p* = 0.253, *q* = 0.502, *ω* = 12.803

Estimated proportions of sites (*p*_x_) evolving at corresponding estimated rates (*ω*_x_ = *d*_N_/*d*_S_) are given in the parameters column.

^†^Alternative models of codon evolution from PAML (Yang 1997, 2007).

^§^Log likelihood score.

^φ^Proportion of sites (*p*) evolving at corresponding rate (*ω*).

^*^Model of best fit.

### Methods for testing evolutionary models

Using maximum likelihood methods implemented in codeML within the PAML 4.8 software package (Yang 1997, 2007), we compared 5 codon‐based models of sequence evolution (MO, M1a, M2a, M7, and M8). These methods gave us an estimate of the selection parameter, *ω*, which measures the ratio between nonsynonymous (*d*_N_) and synonymous (*d*_S_) substitutions per site. This ratio is typically interpreted in a similar way as the *d*_N_/*d*_S_ ratio; that is, a ratio of *ω *> 1 (an excess of nonsynonymous substitutions relative to synonymous substitutions) is typically interpreted as indicative of positive selection, whereas a ratio of *ω *< 1 indicates purifying selection, and a ratio of *ω *= 0 results in a failure to reject neutrality at the codons in question. The MO model assumes 1 rate (1 *ω* ratio) for all sites; the nearly neutral model, M1a, assumes a proportion (*p*_o_) of sites evolving at *ω *< 1 and the rest (*p*_1_) at *ω*_1_ = 1; and the selection model, M2a, adds an additional class of sites to the M1a model that are evolving at *ω *> 1, where *ω* is estimated from the data. M7 and M8 are extensions of M1a and M2a that include variation in *ω* according to a beta‐distributed pattern of substitution rates. The best‐fitting model was chosen on the basis of likelihood ratio tests (LRTs) and Akaike's information criterion (AIC—(Posada and Buckley 2004; Sullivan and Joyce 2005). Two LRTs of positive selection were conducted, as recommended by the PAML developers, comparing M1a versus M2a, and M7 versus M8. Both methods agreed on the choice of the best‐fitting model in all cases. If models incorporating selection fit our data better than their neutral counterparts, then positively selected codons were identified through the Bayes empirical Bayes procedure, also implemented in codeML within PAML (Zhang et al. 2005).

## Appendix 6

Genetic diversity of *Cynomys gunnisoni* at 12 microsatellites used in this study. *T*_a_ is the annealing temperature in PCR; population identifiers (AV2006, SE, ES_C_, AV_C_) are provided in Appendix 1. Allelic richness was weighted according to the smallest sample size (*n *=* *16 from AV2006). The MHC *DRB1* locus is included for comparison.

**Table:**

		# Alleles (w/richness)				*H*_O_ (*H*_E_)				*F*_IS_			
Locus	*T*_a_ (°C)	AV	SE	ES_C_	AV_C_	AV	SE	ES_C_	AV_C_	AV	SE	ES_C_	AV_C_
aA2	55	4 (4)	3 (2.9)	5 (4.8)	4 (3.5)	0.56 (0.59)	0.61 (0.52)	0.63 (0.66)	0.47 (0.42)	−0.118	−0.133	−0.033	−0.082
aA104	51	6 (6)	6 (5.8)	6 (5.7)	6 (5.7)	0.75 (0.73)	0.77 (0.69)	0.83 (0.74)	0.90 (0.74)	0.016	−0.087	−0.093	−0.192
aA111	55	2 (2)	2 (2)	3 (2.9)	3 (2.8)	0.68 (0.48)	0.39 (0.46)	0.43 (0.54)	0.47 (0.52)	0.266	0.031	−0.017	0.071
aD2	51	5 (5)	5 (4.8)	5 (4.4)	4 (3.9)	0.50 (0.65)	0.66 (0.66)	0.73 (0.72)	0.57 (0.59)	0.167	−0.041	0.151	−0.322
aD12	59	4 (4)	3 (2.8)	4 (3.4)	4 (3.5)	0.43 (0.41)	0.11 (0.11)	0.30 (0.31)	0.30 (0.26)	0.211	0.227	0.151	−0.074
aD115	59	6 (6)	4 (3.9)	6 (5.9)	6 (5.8)	0.75 (0.77)	0.61 (0.68)	0.73 (0.74)	0.80 (0.78)	−0.034	−0.199	0.047	0.360
bIGS‐1	55	5 (5)	H5 (4.9)	5 (4.7)	5 (4.9)	0.75 (0.74)	0.44 (0.66)	0.57 (0.64)	0.80 (0.76)	0.060	0.130	0.033	−0.004
bIGS‐6	59	2 (2)	2 (2)	3 (3)	2 (1.9)	0.25 (0.31)	0.22 (0.27)	0.57 (0.65)	0.17 (0.15)	−0.040	−0.015	0.070	−0.101
bIGS‐9d	51	4 (4)	3 (3)	2 (2)	4 (3.6)	0.37 (0.32)	0.61 (0.52)	0.35 (0.32)	0.27 (0.24)	0.022	0.360	0.132	−0.035
bIGS‐24d	59	4 (4)	3 (3)	3 (3)	4 (3.6)	0.50 (0.57)	0.50 (0.46)	0.53 (0.61)	0.73 (0.54)	−0.398	0.185	0.227	0.124
bIGS‐BP1	59	2 (2)	2 (2)	2 (2)	2 (1.9)	0.12 (0.11)	0.61 (0.49)	0.45 (0.46)	0.10 (0.15)	0.094	−0.133	0.063	−0.084
cGS‐14	53	5 (5)	4 (3.9)	4 (3.9)	5 (4.9)	0.93 (0.75)	0.55 (0.58)	0.67 (0.64)	0.73 (0.73)	−0.203	0.079	−0.010	0.022
Mean		4.1 (4.1)	3.4 (3.3)	3.9 (3.8)	4.1 (3.8)	0.55 (0.54)	0.51 (0.51)	0.57 (0.59)	0.52 (0.50)	0.010	0.037	0.059	−0.044
± SE		0.4	0.4	0.4	0.4	0.07 (0.06)	0.05 (0.05)	0.05 (0.04)	0.08 (0.07)				
													
MHC *DRB1*		4	3 (2.9)	6 (5.5)	3 (2.9)	0.50 (0.49)	0.28 (0.25)	0.53 (0.52)	0.27 (0.31)	0.004	−0.090	−0.007	0.164

^a^Jones et al. (2005), ^b^May et al. (1997), ^c^Stevens et al. (1997), ^H^Out of Hardy‐Weinberg equilibrium (*P* = 0.01).

These microsatellite markers were multiplexed according annealing temperature, except markers D2 and GS‐14 were run singly. The PCRs for 11 of the markers were carried out in 10 *μ*L volumes containing 2 *μ*L of DNA template (20 ng/*μ*L), 1 *μ*L 10X PCR buffer, 2 mM MgCl_2_, 0.2 mM dNTPs, 0.08U Platinum^®^ *Taq* polymerase (Invitrogen, Carlsbad, CA), and 0.1 *μ*M**–**0.4 *μ*M of each primer. The PCR profile for these 11 markers started with an antibody releasing step of 95°C for 10 min, followed by 34 cycles of the following: 94°C for 1 min, *T*_a_ (from Table) for 30 sec, and 72°C for 30 sec, with a final extension step of 72°C for 5 min. The PCR for the remaining locus (GS‐14) contained 1.5 mM MgCl_2_, 0.12 mM dNTPs, and 0.2 *μ*M of each primer and the PCR profile followed that of Stevens et al. (1997), except for a 95°C hot start for 10 min and an annealing temperature of 53°C.

## Appendix 7

Frequencies of the six alleles at the MHC class II *DRB1* locus (*MhcCygu‐DRB1*) for all *Cynomys gunnisoni* population samples in this study. The *DRB1* genotype of each individual is provided in Appendix 1.

## Appendix 8

Results of bottleneck tests based on heterozygote excess and the mode shift method (Cornuet and Luikart 1996; Luikart and Cornuet 1998) for 12 microsatellite loci in *Cynomys gunnisoni*. Ratios show the number of microsatellite loci exhibiting heterozygote deficiency vs. excess, and *P*‐values are given for 2‐tailed Wilcoxon signed‐rank tests that evaluate deviations from a 50:50 ration of heterozygosity deficiency:excess (significant *P*‐values are in bold font). Mutational models include the stepwise mutation model (SMM) and infinite alleles model (IAM). For the mode shift test, “L‐shaped” indicates that rare allele frequencies fit a normal L‐shaped distribution expected for a nonbottlenecked population.

**Table:**

Test	Model	AV	SE	ES_C_	AV_C_
*H*_E_ excess	SMM	7:5	6:6	3:9	6:6
		0.733	0.733	**0.092**	0.518
					
	IAM	3:9	1:11	1:11	5:7
		0.176	**0.034**	**0.002**	0.233
					
Mode shift		L‐shaped	L‐shaped	L‐shaped	L‐shaped

## References

[ece32077-bib-0001] Allender, C. J. , W. R. Easterday , M. N. Van Ert , D. M. Wagner , and P. Keim . 2004 High‐throughput extraction of arthropod vector and pathogen DNA using bead milling. Biotechniques 37:730–734. 15560126 10.2144/04375BM03

[ece32077-bib-0002] Altermatt, F. , and D. Ebert . 2008 Genetic diversity of *Daphnia magna* populations enhances resistance to parasites. Ecol. Lett. 11:918–928. 18479453 10.1111/j.1461-0248.2008.01203.x

[ece32077-bib-0003] Altizer, S. , D. Harvell , and E. Friedle . 2003 Rapid evolutionary dynamics and disease threats to biodiversity. Trends Ecol. Evol. 18:589–596.

[ece32077-bib-0004] Anderson, R. M. , and R. M. May . 1979 Population biology of infectious diseases: Part I. Nature 280:361–367. 460412 10.1038/280361a0

[ece32077-bib-0005] Anderson, S. H. , and E. S. Williams . 1997 Plague in a complex of white‐tailed prairie dogs and associated small mammals in Wyoming. J. Wildl. Dis. 33:720–732. 9391955 10.7589/0090-3558-33.4.720

[ece32077-bib-0006] Anisimova, M. , R. Nielsen , and Z. H. Yang . 2003 Effect of recombination on the accuracy of the likelihood method for detecting positive selection at amino acid sites. Genetics 164:1229–1236. 12871927 10.1093/genetics/164.3.1229 PMC1462615

[ece32077-bib-0007] Ari, T. B. , A. Gershunov , R. Tristan , B. Cazelles , K. Gage , and N. C. Stenseth . 2010 Interannual variability of human plague occurrence in the western United States explained by tropical and north Pacific Ocean climate variability. Am. J. Trop. Med. Hyg. 83:624–632. 20810830 10.4269/ajtmh.2010.09-0775 PMC2929061

[ece32077-bib-0008] Babik, W. 2010 Methods for MHC genotyping in non‐model vertebrates. Mol. Ecol. Resour. 10:237–251. 21565019 10.1111/j.1755-0998.2009.02788.x

[ece32077-bib-0009] Beaumont, M. A. , and R. A. Nichols . 1996 Evaluating loci for use in the genetic analysis of population structure. Proc. R. Soc. B Biol. Sci. 263:1619–1626.

[ece32077-bib-0010] de Bellocq, J. G. , N. Charbonnel , and S. Morand . 2008 Coevolutionary relationship between helminth diversity and MHC class II polymorphism in rodents. J. Evol. Biol. 21:1144–1150. 18462313 10.1111/j.1420-9101.2008.01538.x

[ece32077-bib-0011] Biedrzycka, A. , and J. Radwan . 2008 Population fragmentation and major histocompatibility complex variation in the spotted suslik, *Spermophilus suslicus* . Mol. Ecol. 17:4801–4811. 19140973 10.1111/j.1365-294X.2008.03955.x

[ece32077-bib-0012] Biedrzycka, A. , A. Kloch , M. Buczek , and J. Radwan . 2011 Major histocompatibility complex DRB genes and blood parasite loads in fragmented populations of the spotted suslik *Spermophilus suslicus* . Mamm. Biol. 76:672–677.

[ece32077-bib-0013] Biggins, D.E. , J.L. Godbey , M.R. Matchett , and T.M. Livieri . 2006 Habitat preferences and intraspecific competition in black‐footed ferrets. In: Recovery of the black‐footed ferret: progress and continuing challenges. Proceedings of the symposium on the status of the Black‐footed Ferret and its habitat, Fort Collins, CO, January 28‐29, 2004. Scientific Investigations Report 2005‐5293. U.S. Geological Survey 129–140 p.

[ece32077-bib-0014] Blanchet, C. , J. Jaubert , E. Carniel , C. Fayolle , G. Milon , M. Szatanik , et al. 2011 *Mus spretus* SEG/Pas mice resist virulent *Yersinia pestis*, under multigenic control. Genes Immun. 12:23–30. 20861861 10.1038/gene.2010.45

[ece32077-bib-0015] Bodmer, W. F. 1972 Evolutionary significance of the HL‐A system. Nature 237:139–145. 4113158 10.1038/237139a0

[ece32077-bib-0016] Bondinas, G. P. , A. K. Moustakas , and G. K. Papadopoulos . 2007 The spectrum of HLA‐DQ and HLA‐DR alleles, 2006: a listing correlating sequence and structure with function. Immunogenetics 59:539–553. 17497145 10.1007/s00251-007-0224-8

[ece32077-bib-0017] Bosio, C. F. , C. O. Jarrett , D. Gardner , and B. J. Hinnebusch . 2012 Kinetics of innate immune response to *Yersinia pestis* after intradermal infection in a mouse model. Infect. Immun. 80:4034–4045. 22966041 10.1128/IAI.00606-12 PMC3486050

[ece32077-bib-0018] Boyce, W. M. , P. W. Hedrick , N. E. MuggliCockett , S. Kalinowski , M. C. T. Penedo , and R. R. I. Ramey . 1997 Genetic variation of major histocompatibility complex and microsatellite loci: a comparison in bighorn sheep. Genetics 145:421–433. 9071595 10.1093/genetics/145.2.421 PMC1207806

[ece32077-bib-0019] Busch, J. D. , R. Van Andel , J. Cordova , R. E. Colman , P. Keim , T. E. Rocke , et al. 2011 Population differences in host immune factors may influence survival of Gunnison's prairie dogs (*Cynomys gunnisoni*) during plague outbreaks. J. Wildl. Dis. 47:968–973. 22102668 10.7589/0090-3558-47.4.968

[ece32077-bib-0020] Busch, J. D. , R. Van Andel , N. E. Stone , K. R. Cobble , R. Nottingham , J. Lee , et al. 2013 The innate immune response may be important for surviving plague in wild Gunnison's prairie dogs. J. Wildl. Dis. 49:920–931. 24502719 10.7589/2012-08-209

[ece32077-bib-0021] Califf, K. J. , E. K. Ratzloff , A. P. Wagner , K. E. Holekamp , and B. L. Williams . 2013 Forces shaping major histocompatibility complex evolution in two hyena species. J. Mammal. 94:282–294.

[ece32077-bib-0022] Carnero‐Montoro, E. , L. Bonet , J. Engelken , T. Bielig , M. Martinez‐Florensa , F. Lozano , et al. 2012 Evolutionary and functional evidence for positive selection at the human CD5 immune receptor gene. Mol. Biol. Evol. 29:811–823. 21998275 10.1093/molbev/msr251

[ece32077-bib-0023] Castellanos‐Morales, G. , J. Ortega , R. A. Castillo‐Gamez , L. C. Sackett , and L. E. Eguiarte . 2015 Genetic variation and structure in contrasting geographic distributions: widespread versus restricted black‐tailed prairie dogs (subgenus Cynomys). J. Hered. 106(Suppl 1):478–490. 26245783 10.1093/jhered/esv021

[ece32077-bib-0024] Chevallier, L. , C. Blanchet , J. Jaubert , E. Pachulec , C. Demeure , E. Carniel , et al. 2013 Resistance to plague of *Mus spretus* SEG/Pas mice requires the combined action of at least four genetic factors. Genes Immun. 14:35–41. 23151488 10.1038/gene.2012.50

[ece32077-bib-0025] Cornuet, J. M. , and G. Luikart . 1996 Description and power analysis of two tests for detecting recent population bottlenecks from allele frequency data. Genetics 144:2001–2014. 8978083 10.1093/genetics/144.4.2001 PMC1207747

[ece32077-bib-0026] Cui, Y. , C. Yu , Y. Yan , D. Li , Y. Li , T. Jombart , et al. 2013 Historical variations in mutation rate in an epidemic pathogen, *Yersinia pestis* . Proc. Natl Acad. Sci. USA 110:577–582. 23271803 10.1073/pnas.1205750110 PMC3545753

[ece32077-bib-0027] Cully, J. F., Jr . 1997 Growth and life‐history changes in Gunnison's prairie dogs after a plague epizootic. J. Mammal. 78:146–157.

[ece32077-bib-0028] Cully, J. F., Jr , and E. S. Williams . 2001 Interspecific comparisons of sylvatic plague in prairie dogs. J. Mammal. 82:894–905.

[ece32077-bib-0029] Cully, J. F., Jr , A. M. Barnes , T. J. Quan , and G. Maupin . 1997 Dynamics of plague in a Gunnison's prairie dog colony complex from New Mexico. J. Wildl. Dis. 33:706–719. 9391954 10.7589/0090-3558-33.4.706

[ece32077-bib-0030] Cully, J. F., Jr , T. L. Johnson , S. K. Collinge , and C. Ray . 2010 Disease limits populations: plague and black‐tailed prairie dogs. Vector Borne Zoonotic Dis. 10:7–15. 20158327 10.1089/vbz.2009.0045 PMC2945311

[ece32077-bib-0031] Daszak, P. 2000 Emerging infectious diseases of wildlife – Threats to biodiversity and human health. Science 287:1756–1756. 10.1126/science.287.5452.443 10642539

[ece32077-bib-0032] Demeure, C. E. , C. Blanchet , C. Fitting , C. Fayolle , H. Khun , M. Szatanik , et al. 2011 Early systemic bacterial dissemination and a rapid innate immune response characterize genetic resistance to plague of SEG mice. J. Infect. Dis. 205:134–143. 22090450 10.1093/infdis/jir696

[ece32077-bib-0033] Edwards, S. V. , and P. W. Hedrick . 1998 Evolution and ecology of MHC molecules: from genomics to sexual selection. Trends Ecol. Evol. 13:305–311. 21238318 10.1016/s0169-5347(98)01416-5

[ece32077-bib-0150] Erlich, H. A., and T. L. Bugawan. 1990 HLA DNA typing. Pages 261‐271 *in* Innis D. G. M., Sninsky J. and White T., editor. PCR Protocols: A Guide to Methods and Applications. Academic Press, New York, NY.

[ece32077-bib-0034] Eskey, C. R. , and V. H. Haas . 1940 Plague in the western part of the United States Pages 1–83 Public health bulletin. U.S. Public Health Service, Washington, DC.

[ece32077-bib-0035] Figueroa, F. , E. Gunther , and J. Klein . 1988 MHC polymorphism pre‐dating speciation. Nature 335:265–267. 3137477 10.1038/335265a0

[ece32077-bib-0036] Fitak, R. R. , J. L. Koprowski , and M. Culver . 2013 Severe reduction in genetic variation in a montane isolate: the endangered Mount Graham red squirrel (*Tamiasciurus hudsonicus grahamensis*). Conserv. Genet. 14:1233–1241.

[ece32077-bib-0037] Gage, K. L. , and M. Y. Kosoy . 2005 Natural history of plague: perspectives from more than a century of research. Annu. Rev. Entomol. 50:505–528. 15471529 10.1146/annurev.ento.50.071803.130337

[ece32077-bib-0038] Gasper, P. W. , and R. W. Watson . 2001 Plague and yersiniosis Pp. 313–329 *in* Williams E. S. and Barker I. K., eds. Infectious diseases of wild mammals. Iowa State University Press, Ames, IA.

[ece32077-bib-0039] Girard, J. M. , D. M. Wagner , A. J. Vogler , C. Keys , C. J. Allender , L. C. Drickamer , et al. 2004 Differential plague‐transmission dynamics determine *Yersinia pestis* population genetic structure on local, regional, and global scales. Proc. Natl Acad. Sci. USA 101:8408–8413. 15173603 10.1073/pnas.0401561101 PMC420407

[ece32077-bib-0040] Goodwin, H. T. 1995 Pliocene‐Pleistocene biogeographic history of prairie dogs, genus *Cynomys* (Sciuridae). J. Mammal. 76:100–122.

[ece32077-bib-0041] Goudet, J. 1995 FSTAT (Version 1.2): a computer program to calculate F‐statistics. J. Hered. 86:485–486.

[ece32077-bib-0042] Goudet, J . 2001 FSTAT, a program to estimate and test gene diversities and fixation indices (version 2.9.3). Lausanne University, Lausanne, Switzerland.

[ece32077-bib-0043] Griffin, K. A. , D. J. Martin , L. E. Rosen , M. A. Sirochman , D. P. Walsh , L. L. Wolfe , et al. 2010 Detection of *Yersinia pestis* DNA in prairie dog‐associated fleas by polymerase chain reaction assay of purified DNA. J. Wildl. Dis. 46:636–643. 20688665 10.7589/0090-3558-46.2.636

[ece32077-bib-0044] de Groot, N. G. , N. Otting , G. G. M. Doxiadis , S. S. Balla‐Jhagjhoorsingh , J. L. Heeney , J. J. van Rood , et al. 2002 Evidence for an ancient selective sweep in the MHC class I gene repertoire of chimpanzees. Proc. Natl Acad. Sci. USA 99:11748–11753. 12186979 10.1073/pnas.182420799 PMC129340

[ece32077-bib-0045] Harrington, D. P. , and T. R. Fleming . 1982 A class of rank test procedures for censored survival data. Biometrika 69:553–566.

[ece32077-bib-0046] Hedrick, P. W. 2002 Pathogen resistance and genetic variation at MHC loci. Evolution 56:1902–1908. 12449477 10.1111/j.0014-3820.2002.tb00116.x

[ece32077-bib-0047] Herron, M. D. , T. A. Castoe , and C. L. Parkinson . 2004 Sciurid phylogeny and the paraphyly of Holarctic ground squirrels (*Spermophilus*). Mol. Phylogenet. Evol. 31:1015–1030. 15120398 10.1016/j.ympev.2003.09.015

[ece32077-bib-0048] Hill, A. 1991 HLA associations with malaria in Africa: some implications for MHC evolution *in* Klein J. and Klein D., eds. Molecular evolution of the major histocompatibility complex. Springer‐Verlag, Berlin.

[ece32077-bib-0049] Hoogland, J. L. , S. Davis , S. Benson‐Amram , D. Labruna , B. Goossens , and M. A. Hoogland . 2004 Pyraperm kills fleas and halts plague among Utah prairie dogs. Southwest. Nat. 49:376–383.

[ece32077-bib-0050] Jones, R. T. , A. P. Martin , A. J. Mitchell , S. K. Collinge , and C. Ray . 2005 Characterization of 14 polymorphic microsatellite markers for the black‐tailed prairie dog (*Cynomys ludovicianus*). Mol. Ecol. Notes 5:71–73.

[ece32077-bib-0051] Kaufman, J. , J. Salomonsen , and M. Flajnik . 1994 Evolutionary conservation of MHC class I and class II molecules – Different yet the same. Semin. Immunol. 6:411–424. 7654997 10.1006/smim.1994.1050

[ece32077-bib-0052] Keesing, F. , L. K. Belden , P. Daszak , A. Dobson , C. D. Harvell , R. D. Holt , et al. 2010 Impacts of biodiversity on the emergence and transmission of infectious diseases. Nature 468:647–652. 21124449 10.1038/nature09575 PMC7094913

[ece32077-bib-0053] Keller, L. F. , and D. M. Waller . 2002 Inbreeding effects in wild populations. Trends Ecol. Evol. 17:230–241.

[ece32077-bib-0054] Klein, J. 1987 Origin of major histocompatibility complex polymorphism: the trans‐species hypothesis. Hum. Immunol. 19:155–162. 3305436 10.1016/0198-8859(87)90066-8

[ece32077-bib-0055] Klein, J. , and F. Figueroa . 1986 Evolution of the major histocompatibility complex. Crit. Rev. Immunol. 6:295–386. 3536303

[ece32077-bib-0056] Klein, J. , R. E. Bontrop , R. L. Dawkins , H. A. Erlich , U. B. Gyllensten , E. R. Heise , et al. 1990 Nomenclature for the major histocompatibility complexes of different species: a proposal. Immunogenetics 31:217–219. 2329006 10.1007/BF00204890

[ece32077-bib-0057] Klein, J. , Y. Satta , C. Ohuigin , and N. Takahata . 1993 The molecular descent of the major histocompatibility complex. Annu. Rev. Immunol. 11:269–295. 8476562 10.1146/annurev.iy.11.040193.001413

[ece32077-bib-0058] Kloch, A. , K. Baran , M. Buczek , M. Konarzewski , and J. Radwan . 2013 MHC influences infection with parasites and winter survival in the root vole *Microtus oeconomus* . Evol. Ecol. 27:635–653.

[ece32077-bib-0059] Kryazhimskiy, S. , and J. B. Plotkin . 2008 The population genetics of dN/dS. PLoS Genet. 4:e1000304. 19081788 10.1371/journal.pgen.1000304 PMC2596312

[ece32077-bib-0060] Kuduk, K. , A. Johanet , D. Allaine , A. Cohas , and J. Radwan . 2012 Contrasting patterns of selection acting on MHC class I and class II *DRB* genes in the Alpine marmot (*Marmota marmota*). J. Evol. Biol. 25:1686–1693. 22594882 10.1111/j.1420-9101.2012.02537.x

[ece32077-bib-0061] Landry, C. , and L. Bernatchez . 2001 Comparative analysis of population structure across environments and geographical scales at major histocompatibility complex and microsatellite loci in Atlantic salmon (*Salmo salar*). Mol. Ecol. 10:2525–2539. 11742552 10.1046/j.1365-294x.2001.01383.x

[ece32077-bib-0062] Librado, P. , and J. Rozas . 2009 DnaSP v5: a software for comprehensive analysis of DNA polymorphism data. Bioinformatics 25:1451–1452. 19346325 10.1093/bioinformatics/btp187

[ece32077-bib-0063] Link, V. B. 1955 A history of plague in the United States of America. Public Health Monogr. 26:1–120. 14371919

[ece32077-bib-0064] Luikart, G. , and J. M. Cornuet . 1998 Empirical evaluation of a test for identifying recently bottlenecked populations from allele frequency data. Conserv. Biol. 12:228–237.

[ece32077-bib-0065] Luikart, G. , F. W. Allendorf , J. M. Cornuet , and W. B. Sherwin . 1998 Distortion of allele frequency distributions provides a test for recent population bottlenecks. J. Hered. 89:238–247. 9656466 10.1093/jhered/89.3.238

[ece32077-bib-0066] Maruyama, T. , and P. A. Fuerst . 1985 Population bottlenecks and nonequilibrium models in population genetics. 2. Number of alleles in a small population that was formed by a recent bottleneck. Genetics 111:675–689. 4054612 10.1093/genetics/111.3.675 PMC1202664

[ece32077-bib-0067] May, B. , T. A. Gavin , P. W. Sherman , and T. M. Korves . 1997 Characterization of microsatellite loci in the northern Idaho ground squirrel *Spermophilus brunneus brunneus* . Mol. Ecol. 6:399–400. 9131817 10.1046/j.1365-294x.1997.00203.x

[ece32077-bib-0068] Mencher, J. S. , S. R. Smith , T. D. Powell , D. T. Stinchcomb , J. E. Osorio , and T. E. Rocke . 2004 Protection of black‐tailed prairie dogs (*Cynomys ludovicianus*) against plague after voluntary consumption of baits containing recombinant raccoon poxvirus vaccine. Infect. Immun. 72:5502–5505. 15322054 10.1128/IAI.72.9.5502-5505.2004 PMC517477

[ece32077-bib-0069] Miller, H. C. , and D. M. Lambert . 2004 Genetic drift outweighs balancing selection in shaping post‐bottleneck major histocompatibility complex variation in New Zealand robins (Petroicidae). Mol. Ecol. 13:3709–3721. 15548285 10.1111/j.1365-294X.2004.02368.x

[ece32077-bib-0070] Moreno‐Cugnon, L. , A. Esparza‐Baquer , A. Larruskain , K. Garcia‐Etxebarria , S. Menne , G. Gonzalez‐Aseguinolaza , et al. 2015 Characterization and genotyping of the DRB1 gene of the major histocompatibility complex (MHC) in the *Marmota monax*, animal model of hepatitis B. Mol. Immunol. 63:505–512. 25458311 10.1016/j.molimm.2014.10.011

[ece32077-bib-0071] Nei, M. , and T. Gojobori . 1986 Simple methods for estimating the numbers of synonymous and nonsynonymous nucleotide substitutions. Mol. Biol. Evol. 3:418–426. 3444411 10.1093/oxfordjournals.molbev.a040410

[ece32077-bib-0072] O'Brien, S. J. , and J. F. Evermann . 1988 Interactive influence of infectious‐disease and genetic diversity in natural populations. Trends Ecol. Evol. 3:254–259. 21227241 10.1016/0169-5347(88)90058-4 PMC7134056

[ece32077-bib-0073] Parent, M. A. , K. N. Berggren , I. K. Mullarky , F. M. Szaba , L. W. Kummer , J. J. Adamovicz , et al. 2005 *Yersinia pestis* V protein epitopes recognized by CD4 T cells. Infect. Immun. 73:2197–2204. 15784563 10.1128/IAI.73.4.2197-2204.2005 PMC1087457

[ece32077-bib-0074] Paterson, S. , K. Wilson , and J. M. Pemberton . 1998 Major histocompatibility complex variation associated with juvenile survival and parasite resistance in a large unmanaged ungulate population (*Ovis aries* L.). Proc. Natl Acad. Sci. USA 95:3714–3719. 9520432 10.1073/pnas.95.7.3714 PMC19902

[ece32077-bib-0075] Pauli, J. N. , S. W. Buskirk , E. S. Williams , and W. H. Edwards . 2006 A plague epizootic in the black‐tailed prairie dog (*Cynomys ludovicianus*). J. Wildl. Dis. 42:74–80. 16699150 10.7589/0090-3558-42.1.74

[ece32077-bib-0076] Piry, S. , G. Luikart , and J. M. Cornuet . 1999 BOTTLENECK: a computer program for detecting recent reductions in the effective population size using allele frequency data. J. Hered. 90:502–503.

[ece32077-bib-0077] Posada, D. , and T. Buckley . 2004 Model selection and model averaging in phylogenetics: advantages of akaike information criterion and bayesian approaches over likelihood ratio tests. Syst. Biol. 53:793–808. 15545256 10.1080/10635150490522304

[ece32077-bib-0078] Potts, W. K. , and E. K. Wakeland . 1990 Evolution of diversity at the major histocompatibility complex. Trends Ecol. Evol. 5:181–187. 21232350 10.1016/0169-5347(90)90207-T

[ece32077-bib-0079] Quan, S. F. , and L. Kartman . 1962 Ecological studies of wild rodent plague in the San Francisco Bay area of California. VIII. Susceptibility of wild rodents to experimental plague infection. Zoonoses Res. 1:121–144. 14489383

[ece32077-bib-0080] Quan, T. J. , A. M. Barnes , L. G. Carter , and K. R. Tsuchiya . 1985 Experimental plague in rock squirrels, *Spermophilus variegatus* (Erxleben). J. Wildl. Dis. 21:205–210. 4032620 10.7589/0090-3558-21.3.205

[ece32077-bib-0081] Rasmussen, S. , M. E. Allentoft , K. Nielsen , L. Orlando , M. Sikora , K. G. Sjogren , et al. 2015 Early divergent strains of *Yersinia pestis* in Eurasia 5000 years ago. Cell 163:571–582. 26496604 10.1016/j.cell.2015.10.009 PMC4644222

[ece32077-bib-0082] Reid, N. , S. Hird , A. Schulte‐Hostedde , and J. Sullivan . 2010 Examination of nuclear loci across a zone of mitochondrial introgression between *Tamias ruficaudus* and *T. amoenus* . J. Mammal. 91:1389–1400.

[ece32077-bib-0083] Ricanova, S. , J. Bryja , J. F. Cosson , G. Csongor , L. Choleva , M. Ambros , et al. 2011 Depleted genetic variation of the European ground squirrel in Central Europe in both microsatellites and the major histocompatibility complex gene: implications for conservation. Conserv. Genet. 12:1115–1129.

[ece32077-bib-0084] Roach, J. L. , P. Stapp , B. Van Horne , and M. F. Antolin . 2001 Genetic structure of a metapopulation of black‐tailed prairie dogs. J. Mammal. 82:946–959.

[ece32077-bib-0085] Rocke, T. E. , S. R. Smith , D. T. Stinchcomb , and J. E. Osorio . 2008 Immunization of black‐tailed prairie dog against plague through consumption of vaccine‐laden baits. J. Wildl. Dis. 44:930–937. 18957649 10.7589/0090-3558-44.4.930

[ece32077-bib-0086] Rocke, T. E. , N. Pussini , S. R. Smith , J. Williamson , B. Powell , and J. E. Osorio . 2010 Consumption of baits containing raccoon pox‐based plague vaccines protects black‐tailed prairie dogs (*Cynomys ludovicianus*). Vector Borne Zoonotic Dis. 10:53–58. 20158332 10.1089/vbz.2009.0050

[ece32077-bib-0087] Rocke, T. E. , J. Williamson , K. R. Cobble , J. D. Busch , M. F. Antolin , and D. M. Wagner . 2012 Resistance to plague among black‐tailed prairie dog populations. Vector Borne Zoonotic Dis. 12:111–116. 21923261 10.1089/vbz.2011.0602 PMC3267546

[ece32077-bib-0088] Roemer, D. M. , and S. C. Forrest . 1996 Prairie dog poisoning in northern Great Plains: an analysis of programs and policies. Environ. Manage. 20:349–359. 8661606 10.1007/BF01203843

[ece32077-bib-0089] Rust, J. H. , D. N. Harrison , J. D. Marshall, Jr , and D. C. Cavanaugh . 1972 Susceptibility of rodents to oral plague infection: a mechanism for the persistence of plague in inter‐epidemic periods. J. Wildl. Dis. 8:127–133. 5020998 10.7589/0090-3558-8.2.127

[ece32077-bib-0090] Sackett, L. C. , T. B. Cross , R. T. Jones , W. C. Johnson , K. Ballare , C. Ray , et al. 2012 Connectivity of prairie dog colonies in an altered landscape: inferences from analysis of microsatellite DNA variation. Conserv. Genet. 13:407–418.

[ece32077-bib-0091] Sackett, L. C. , S. K. Collinge , and A. P. Martin . 2013 Do pathogens reduce genetic diversity of their hosts? Variable effects of sylvatic plague in black‐tailed prairie dogs. Mol. Ecol. 22:2441–2455. 23452304 10.1111/mec.12270

[ece32077-bib-0092] Sackett, L. C. , A. Seglund , R. P. Guralnick , M. N. Mazzella , D. M. Wagner , J. D. Busch , et al. 2014 Evidence for two subspecies of Gunnison's prairie dogs (*Cynomys gunnisoni*), and the general importance of the subspecies concept. Biol. Conserv. 174:1–11.

[ece32077-bib-0093] Sena, L. , M. P. C. Schneider , B. Brenig , R. L. Honeycutt , J. E. Womack , and L. C. Skow . 2003 Polymorphisms in MHC‐DRA and ‐DRB alleles of water buffalo (*Bubalus bubalis*) reveal different features from cattle DR alleles. Anim. Genet. 34:1–10. 12580780 10.1046/j.1365-2052.2003.00920.x

[ece32077-bib-0094] Shim, H.‐K. , J. A. Musson , H. M. Harper , H. V. McNeill , N. Walker , H. Flick‐Smith , et al. 2006 Mechanisms of major histocompatibility complex class II‐restricted processing and presentation of the V antigen of *Yersinia pestis* . Immunology 119:385–392. 16919002 10.1111/j.1365-2567.2006.02447.x PMC1819574

[ece32077-bib-0095] Siddle, H. V. , A. Kreiss , M. D. B. Eldridge , E. Noonan , C. J. Clarke , S. Pyecroft , et al. 2007 Transmission of a fatal clonal tumor by biting occurs due to depleted MHC diversity in a threatened carnivorous marsupial. Proc. Natl Acad. Sci. USA 104:16221–16226. 17911263 10.1073/pnas.0704580104 PMC1999395

[ece32077-bib-0096] Slade, R. W. , and H. I. McCallum . 1992 Overdominant vs. frequency‐dependent selection at MHC loci. Genetics 132:861–864. 1468635 10.1093/genetics/132.3.861 PMC1205221

[ece32077-bib-0097] Sommer, S. 2005 The importance of immune gene variability (MHC) in evolutionary ecology and conservation. Front. Zool. 2:16. 16242022 10.1186/1742-9994-2-16 PMC1282567

[ece32077-bib-0098] Soundararajan, V. , N. Patel , V. Subramanian , V. Sasisekharan , and R. Sasisekharan . 2011 The many faces of the YopM effector from plague causative bacterium *Yersinia pestis* and its implications for host immune modulation. Innate Immun. 17:548–557. 20699282 10.1177/1753425910377099

[ece32077-bib-0099] Stevens, S. , J. Coffin , and C. Strobeck . 1997 Microsatellite loci in Columbian ground squirrels *Spermophilus columbianus* . Mol. Ecol. 6:493–495. 9161017 10.1046/j.1365-294x.1997.t01-1-00192.x

[ece32077-bib-0100] Strand, T. M. , G. Segelbacher , M. Quintela , L. Y. Xiao , T. Axelsson , and J. Hoglund . 2012 Can balancing selection on MHC loci counteract genetic drift in small fragmented populations of black grouse? Ecol. Evol. 2:341–353. 22423328 10.1002/ece3.86 PMC3298947

[ece32077-bib-0101] Sullivan, J. , and P. Joyce . 2005 Model selection in phylogenetics. Annu. Rev. Ecol. Evol. Syst. 36:445–466.

[ece32077-bib-0102] Sutton, J. T. , B. C. Robertson , and I. G. Jamieson . 2015 MHC variation reflects the bottleneck histories of New Zealand passerines. Mol. Ecol. 24:362–373. 25488544 10.1111/mec.13039

[ece32077-bib-0103] Takahata, N. , and M. Nei . 1990 Allelic genealogy under overdominant and frequency‐dependent selection and polymorphism of major histocompatibility complex loci. Genetics 124:967–978. 2323559 10.1093/genetics/124.4.967 PMC1203987

[ece32077-bib-0104] Tamura, K. , G. Stecher , D. Peterson , A. Filipski , and S. Kumar . 2013 MEGA6: molecular evolutionary genetics analysis version 6.0. Mol. Biol. Evol. 30:2725–2729. 24132122 10.1093/molbev/mst197 PMC3840312

[ece32077-bib-0105] Thomas, R. E. , A. M. Barnes , T. J. Quan , M. L. Beard , L. G. Carter , and C. E. Hopla . 1988 Susceptibility to *Yersinia pestis* in the northern grasshopper mouse (*Onychomys leucogaster*). J. Wildl. Dis. 24:327–333. 3373638 10.7589/0090-3558-24.2.327

[ece32077-bib-0106] Thomas, R. E. , M. L. Beard , T. J. Quan , L. G. Carter , A. M. Barnes , and C. E. Hopla . 1989 Experimentally induced plague infection in the northern grasshopper mouse (*Onychomys leucogaster*) acquired by consumption of infected prey. J. Wildl. Dis. 25:477–480. 2810547 10.7589/0090-3558-25.4.477

[ece32077-bib-0107] Thoss, M. , P. Ilmonen , K. Musolf , and D. J. Penn . 2011 Major histocompatibility complex heterozygosity enhances reproductive success. Mol. Ecol. 20:1546–1557. 21291500 10.1111/j.1365-294X.2011.05009.x

[ece32077-bib-0108] Tollenaere, C. , J. M. Duplantier , L. Rahalison , M. Ranjalahy , and C. Brouat . 2010a AFLP genome scan in the black rat (*Rattus rattus*) from Madagascar: detecting genetic markers undergoing plague‐mediated selection. Mol. Ecol. 20:1026–1038. 20444082 10.1111/j.1365-294X.2010.04633.x

[ece32077-bib-0109] Tollenaere, C. , L. Rahalison , M. Ranjalahy , J. M. Duplantier , S. Rahelinirina , S. Telfer , et al. 2010b Susceptibility to *Yersinia pestis* experimental infection in wild *Rattus rattus*, reservoir of plague in Madagascar. EcoHealth 7:242–247. 20443044 10.1007/s10393-010-0312-3

[ece32077-bib-0110] Tollenaere, C. , S. Ivanova , J. M. Duplantier , A. Loiseau , L. Rahalison , S. Rahelinirina , et al. 2012 Contrasted patterns of selection on MHC‐linked microsatellites in natural populations of the Malagasy plague reservoir. PLoS One 7:e32814. 22403713 10.1371/journal.pone.0032814 PMC3293896

[ece32077-bib-0111] Tollenaere, C. , S. Jacquet , S. Ivanova , A. Loiseau , J. M. Duplantier , R. Streiff , et al. 2013 Beyond an AFLP genome scan towards the identification of immune genes involved in plague resistance in *Rattus rattus* from Madagascar. Mol. Ecol. 22:354–367. 23237097 10.1111/mec.12115

[ece32077-bib-0112] Turner, J. K. , M. M. McAllister , J. L. Xu , and R. I. Tapping . 2008 The resistance of BALB/cJ mice to *Yersinia pestis* maps to the major histocompatibility complex of chromosome 17. Infect. Immun. 76:4092–4099. 18573896 10.1128/IAI.00488-08 PMC2519398

[ece32077-bib-0113] Van Pelt, W. E. 1995 Assessment of potential black‐footed ferret habitat in northern Arizona. Arizona Game and Fish Department, Phoenix, AZ.

[ece32077-bib-0114] Wagner, D. M. , L. C. Drickamer , D. M. Krpata , C. J. Allender , W. E. Van Pelt , and P. Keim . 2006 Persistence of Gunnison's prairie dog colonies in Arizona, USA. Biol. Conserv. 130:331–339.

[ece32077-bib-0115] Wilson, D. J. , and G. McVean . 2006 Estimating diversifying selection and functional constraint in the presence of recombination. Genetics 172:1411–1425. 16387887 10.1534/genetics.105.044917 PMC1456295

[ece32077-bib-0116] Wolf, J. B. , A. Kunstner , K. Nam , M. Jakobsson , and H. Ellegren . 2009 Nonlinear dynamics of nonsynonymous (dN) and synonymous (dS) substitution rates affects inference of selection. Genome Biol. Evol. 1:308–319. 20333200 10.1093/gbe/evp030 PMC2817425

[ece32077-bib-0117] Worley, K. , J. Carey , A. Veitch , and D. W. Coltman . 2006 Detecting the signature of selection on immune genes in highly structured populations of wild sheep (*Ovis dalli*). Mol. Ecol. 15:623–637. 16499690 10.1111/j.1365-294X.2006.02829.x

[ece32077-bib-0118] Yang, Z. 1997 PAML: a program package for phylogenetic analysis by maximum likelihood. Comput. Appl. Biosci. 13:555–556. 9367129 10.1093/bioinformatics/13.5.555

[ece32077-bib-0151] Yang, Z. 2007 PAML 4: a program package for phylogenetic analysis by maximum likelihood. Mol. Biol. Evol. 24:1586–1591. 17483113 10.1093/molbev/msm088

[ece32077-bib-0119] Yeager, M. , and A. L. Hughes . 1999 Evolution of the mammalian MHC: natural selection, recombination, and convergent evolution. Immunol. Rev. 167:45–58. 10319250 10.1111/j.1600-065x.1999.tb01381.x

[ece32077-bib-0120] Yuhki, N. , J. C. Mullikin , T. Beck , R. Stephens , and S. J. O'Brien . 2008 Sequences, annotation and single nucleotide polymorphism of the major histocompatibility complex in the domestic cat. PLoS One 3:e2674. 18629345 10.1371/journal.pone.0002674 PMC2453318

[ece32077-bib-0121] Zeidner, N. S. , L. G. Carter , J. A. Monteneiri , J. M. Petersen , M. Schriefer , K. L. Gage , et al. 2004 An outbreak of *Francisella tularensis* in captive prairie dogs: an immunohistochemical analysis. J. Vet. Diagn. Invest. 16:150–152. 15053367 10.1177/104063870401600210

[ece32077-bib-0122] Zhang, J. , R. Nielsen , and Z. Yang . 2005 Evaluation of an improved branch‐site likelihood method for detecting positive selection at the molecular level. Mol. Biol. Evol. 22:2472–2479. 16107592 10.1093/molbev/msi237

